# Human hepatocyte-enriched miRNA-192-3p promotes HBV replication through inhibiting Akt/mTOR signalling by targeting ZNF143 in hepatic cell lines

**DOI:** 10.1080/22221751.2022.2037393

**Published:** 2022-02-21

**Authors:** Fahong Li, Yingying Deng, Shenyan Zhang, Beidi Zhu, Jun Wang, Jinyu Wang, Xueyu Wang, Zhenyu Zhao, Wanyu Deng, Richeng Mao, Zhongliang Shen, Jieliang Chen, Ruth Broering, Yong Lin, Mengji Lu, Jiming Zhang

**Affiliations:** a Department of Infectious Diseases, Shanghai Key Laboratory of Infectious Diseases and Biosafety Emergency Response, Shanghai Institute of Infectious Diseases and Biosecurity, National Medical Center for Infectious Diseases, Huashan Hospital, Fudan University, Shanghai, People’s Republic of China; b Institute for Virology, University Hospital Essen, University of Duisburg-Essen, Essen, Germany; c Key Laboratory of Molecular Biology of Infectious Diseases (Chinese Ministry of Education), Chongqing Medical University, Chongqing, People’s Republic of China; d Department of Biliary Pancreatic Surgery, Sun Yat-sen Memorial Hospital, Sun Yat-sen University, Guangzhou, Guangdong, People’s Republic of China; e Key Laboratory of Medical Molecular Virology (MOE/MOH), Shanghai Medical College, Fudan University, Shanghai, People’s Republic of China; f Department of Gastroenterology and Hepatology, University Hospital Essen, University of Duisburg-Essen, Essen, Germany; g Department of Infectious Diseases, Jing’An Branch of Huashan Hospital, Fudan University, Shanghai, People’s Republic of China

**Keywords:** MiRNA profile, miR-192-3p, hepatitis B virus, ZNF143, Akt/mTOR signalling, Abbreviations: CMIA: chemiluminescent microparticle immunoassay, HBV: hepatitis B virus, HSC: hepatic stellate cell, KC: Kupffer cell, LSEC: liver sinusoidal endothelial cell, pgRNA: pregenomic RNA, PHH: primary human hepatocyte, RI: replicative intermediate

## Abstract

Previous studies have revealed multiple tissue- or cell-specific or enriched miRNA profiles. However, miRNA profiles enriched in hepatic cell types and their effect on HBV replication have not been well elucidated. In this study, primary human hepatocytes (PHHs), Kupffer cells (KCs), liver sinusoidal endothelial cells (LSECs), and hepatic stellate cells (HSCs) were prepared from liver specimens of non-HBV-infected patients. Four hepatic cell type-enriched miRNA profiles were identified from purified liver cells miRNA microarray assay. The results revealed that 12 miRNAs, including miR-122-5p and miR-192-3p were PHH-enriched; 9 miRNAs, including miR-142-5p and miR-155-5p were KC-enriched; 6 miRNAs, including miR-126-3p and miR-222-3p were LSEC-enriched; and 14 miRNAs, including miR-214-3p and miR-199a-3p were HSC-enriched. By testing the effect of 11 PHH-enriched miRNAs on HBV production, we observed that miR-192-3p had the greatest pro-virus effect in hepatic cell lines. Moreover, we further found that miR-192-3p promoted HBV replication and gene expression through inhibiting Akt/mTOR signalling by direct targeting of ZNF143 in HepG2.2.15 cells. Additionally, the serum and hepatic miR-192-3p expression levels were significantly higher in chronic hepatitis B patients than in healthy controls and serum miR-192-3p positively correlated with the serum levels of HBV DNA and HBsAg. Collectively, we identified miRNA profiles enriched in four hepatic cell types and revealed that PHH-enriched miR-192-3p promoted HBV replication through inhibiting Akt/mTOR signalling by direct targeting of ZNF143 in hepatic cell lines. Our study provides a specific perspective for the role of hepatic cell type-enriched miRNA in interaction with viral replication and various liver pathogenesis.

## Introduction

Approximately 248 million patients are chronically infected with the hepatitis B virus (HBV) worldwide [1]. Chronic HBV infection progresses to liver fibrosis, cirrhosis, or hepatocellular carcinoma. These diseases cause a heavy burden on public health [2]. Recently, many studies based on transcriptome analysis have revealed new aspects of HBV infection in patients and in animal models [3,4]. These studies have provided new insights into molecular and immunological mechanisms of the pathogenesis of various HBV-related liver diseases.

The liver is the largest internal organ, with many functions such as synthesis or degradation of proteins, glycogen, or triglycerides, and detoxification. The liver is composed of primary human hepatocytes (PHHs) and non-parenchymal cells, which include Kupffer cells (KCs) (∼15%), liver sinusoidal endothelial cells (LSECs) (15–20%), and hepatic stellate cells (HSCs) (5–8%), as well as a very small fraction of cholangiocytes (2–3%) [5]. PHHs represent the major cell type of the liver, accounting for about 60–70% of the total liver cell population. Each cell type plays a vital and specific role in liver disease. Hepatocytes, which are responsible for the synthesis of glycogen, protein, and fat, are host cells of HBV and are involved in the development of hepatocellular carcinoma [6].

Cellular miRNAs play vital roles in the regulation of physiological and pathological processes. MicroRNAs are endogenous, ∼22-nucleotide-long, noncoding RNAs that regulate the expression of a large number of genes at the post-transcriptional level [7]. MicroRNAs are distributed in a tissue- and cell-type-specific pattern. Profiling the specific miRNA expression pattern in distinct tissues or cell types provides a basis to understand the actions of miRNAs in various physiological and pathological conditions. Previously, Landgraf et al. established a mammalian miRNA expression atlas; they identified miR-122 as a specific miRNA for the liver, while miR-142, miR-144, miR-150, miR-155, miR-223, and others were identified in haematopoietic cells [8]. Recently, Ludwig et al. further updated the human miRNA tissue atlas [9]. Oda et al. identified the specific miRNA profiles of PHHs and LSECs in rats [10]. However, the four kinds of hepatic cell type-specific or enriched miRNA profiles in humans are still unknown.

Previous studies have reported that many miRNAs are involved in the HBV life cycle and development of HBV-associated liver diseases. A cluster of miRNAs, including miR-122, miR-21, miR-222, miR-223, and miR-199, are associated with hepatocellular carcinoma genesis and development [11]. The miR-133 and miR-101 suppress liver fibrosis by targeting the TGF-β signalling pathway [12]. The miR-29 family is also involved in the development of liver fibrosis [13]. Some specific cellular miRNAs directly interact with HBV mRNAs and regulate HBV gene expression and replication. For example, miR-199a-3p and miR-210 may directly bind to HBV transcripts and reduce HBsAg expression [14]. High expression levels of HBV RNAs may sequester miRNA-122, resulting in an enhanced expression of its target mRNAs [15]. Previously, we reported that miR-1, miR-125b, and miR-99 family regulate viral replication via different mechanisms [16–18].

In the present study, we aimed to identify the four hepatic cell type-enriched miRNA expression profiles. Subsequently, we further investigated the effect of PHH-enriched miRNAs on viral replication and elucidated the potential mechanism of PHH-enriched miR-192-3p in regulating viral replication. Our findings provide a specific perspective for the potential role of hepatic cell type-enriched miRNAs in viral replication and various liver diseases.

## Materials and methods

### Isolation and culture of parenchymal and non-parenchymal liver cells

Fresh, lesion-free liver specimens (25–100 g) were obtained from tumour resections. The study conformed to the ethical guidelines of the 1975 Declaration of Helsinki and was approved by the Institutional Review Board (ethics committee) of the Medical Faculty at the University of Duisburg-Essen. PHHs were seeded at a density of 1.25 to 2.5 × 10^5^ viable cells per cm^2^ onto collagen-I-coated plates (BD Biosciences, USA), using DMEM/Ham’s F-12 medium (Biochrome, Berlin, Germany) supplemented with 10% FBS. KCs were seeded onto plastic culture plates at a density of 4 to 6 × 10^5^ cells per cm^2^ using DMEM supplemented with 10% FBS. LSECs were cultured on collagen-I-coated plates using Endothelial Growth Medium 2 (Promo Cell, Heidelberg, Germany). HSCs were seeded onto uncoated plastic culture flasks using Stellate Cell Medium (ScienCell, Carlsbad, CA, USA). The purity and functionality of cultured cell populations were controlled by determining their morphology, discriminative cell marker expression, and functional activity [19]. As previously reported [19], PHHs, KCs, LSECs, and HSCs were isolated from liver tissues by collagenase perfusion in combination with low-speed centrifugation, density gradient centrifugation, and magnetic-activated cell sorting. Hepatocytes were identified by albumin and exhibited time-dependent activity of cytochrome P450 enzymes. Kupffer cells expressed CD68 and exhibited phagocytic activity, as determined with 1 μm latex beads. Endothelial cells were CD146+ and exhibited efficient uptake of acetylated low-density lipoprotein. Hepatic stellate cells were identified by the expression of α-smooth muscle actin. The clinical characteristics of the included patients are listed in Table S1. PHHs from three, KCs from five, LSECs from two, and HSCs from four patients were sent for further microarray analysis.

### miRNA microarray

Total RNA was extracted and subjected to electrophoresis. RNA Integrity Number was used for standardization of the RNA quality control. Total RNA was extracted and analysed by using Agilent Human miRNA (8 × 60K) array. Agilent Human miRNA microarrays were designed according to miRBase V 21.0, including 2549 human miRNAs. The total signals reported by all of the probes were normalized and the signal value was rescaled on a logarithmic scale (base 2). Fold change between the two groups was calculated as a ratio of the mean raw signal values of each group.

### Blood sampling

A total of 109 serum samples from treatment-naïve chronic hepatitis B (CHB) patients were collected at the outpatient section of the Department of Infectious Disease, Huashan Hospital. The serum samples were processed as described previously [20] and stored at −80°C until use. Twenty age- and gender-matched healthy volunteers were enrolled as healthy controls. The demographic characteristics of the patients are listed in Table S2. HBV DNA was measured quantitatively using a real-time PCR kit (Kehua Bio-engineering Co., Ltd., Shanghai, China), which has a lower limit of detection of 500 IU/mL. Informed written consent was obtained from all patients and the study was approved by the Institutional Ethics Committee for human studies at Huashan Hospital, Fudan University, Shanghai, China. All procedures were in accordance with the Declaration of Helsinki.

### Liver specimens

Liver specimens from three CHB patients with HBV DNA load >10^5^ IU/mL were collected using a 16-gauge Menghini needle. Four normal liver specimens were collected from resection of hepatic haemangioma. Three liver tumour specimens were collected from resection of hepatocellular carcinoma. Half was fixed in formalin, embedded in paraffin for pathological examination, and the remaining half was saved in RNA later and stored at −80°C for further use.

### Plasmids and reagents

pSM2, an HBV replication-competent clone harbouring a head-to-tail tandem dimer of the HBV genome (GenBank accession number V01460, genotype D) was a kind gift from Dr Hans Will (Heinrich-Pette-Institute, Hamburg, Germany). The plasmid with ZNF143 expression was constructed on empty vector p3×FLAG-CMV-10 by General Biol Company (Chuzhou, China). The miRNA mimics, miRNAs inhibitor mimics, and all siRNAs were purchased from Qiagen. The mTOR activator MHY1485 was provided by Selleckchem. The mTOR inhibitor rapamycin (Calbiochem) was purchased from Merck Millipore (Germany).

### Real-time quantitative RT-PCR

Serum and hepatic miRNAs were isolated and quantified using miRcute miRNA Extraction kit (TIANGEN, Beijing, China), first-strand cDNA synthesis, and quantitative PCR detection kits, in accordance with the manufacturer’s instructions. The expression level of mature miRNA was determined by qRT-PCR analysis using commercial miScript Primer or QuantiTect Primer Assays from Qiagen. Total HBV RNA was extracted and detected as described previously [18].

### Luciferase reporter gene assay

The Dual-Glo luciferase reporter assay system (Promega, E2940) was used to detect the firefly luciferase activity and the internal control Renilla luciferase activity, separately. The firefly luciferase reporter plasmids pSP1, pSP2, pCP, and pXP, containing HBV SP1, SP2, core, and X promoters were constructed as previously reported [18]. HBV full-length genome and partial genome fragments were cloned into downstream of the luciferase gene of pMIR-REPORT vectors (Ambion, Austin, TX), generating luciferase reporter pmiR-HBV1, pmiR-HBV2, pmiR-HBV3, and pmiR-HBV 3′UTR. The details of vector construction and luciferase reporter assay were referred in Supplementary Methods and the primers used for cloning are listed in Table S3.

### Statistical analysis

Data were analysed using GraphPad prism 7 (GraphPad Software Inc., San Diego, CA). Statistical significance was determined using the Student’s *t*-test, Kruskal–Wallis test, or Mann–Whitney *t*-test. Spearman’s correlation was used to evaluate the correlation coefficient (*r*). *P*-value <.05 was considered statistically significant.

Cell culture and transfection, analysis of HBV replicative capacity, Western blotting, and effect of miR-192-3p on HBV replication in HBV-infected-primary human hepatocytes are described in Supplementary Methods.

## Results

### Cell type-enriched miRNA profiles

**Figure 1.**
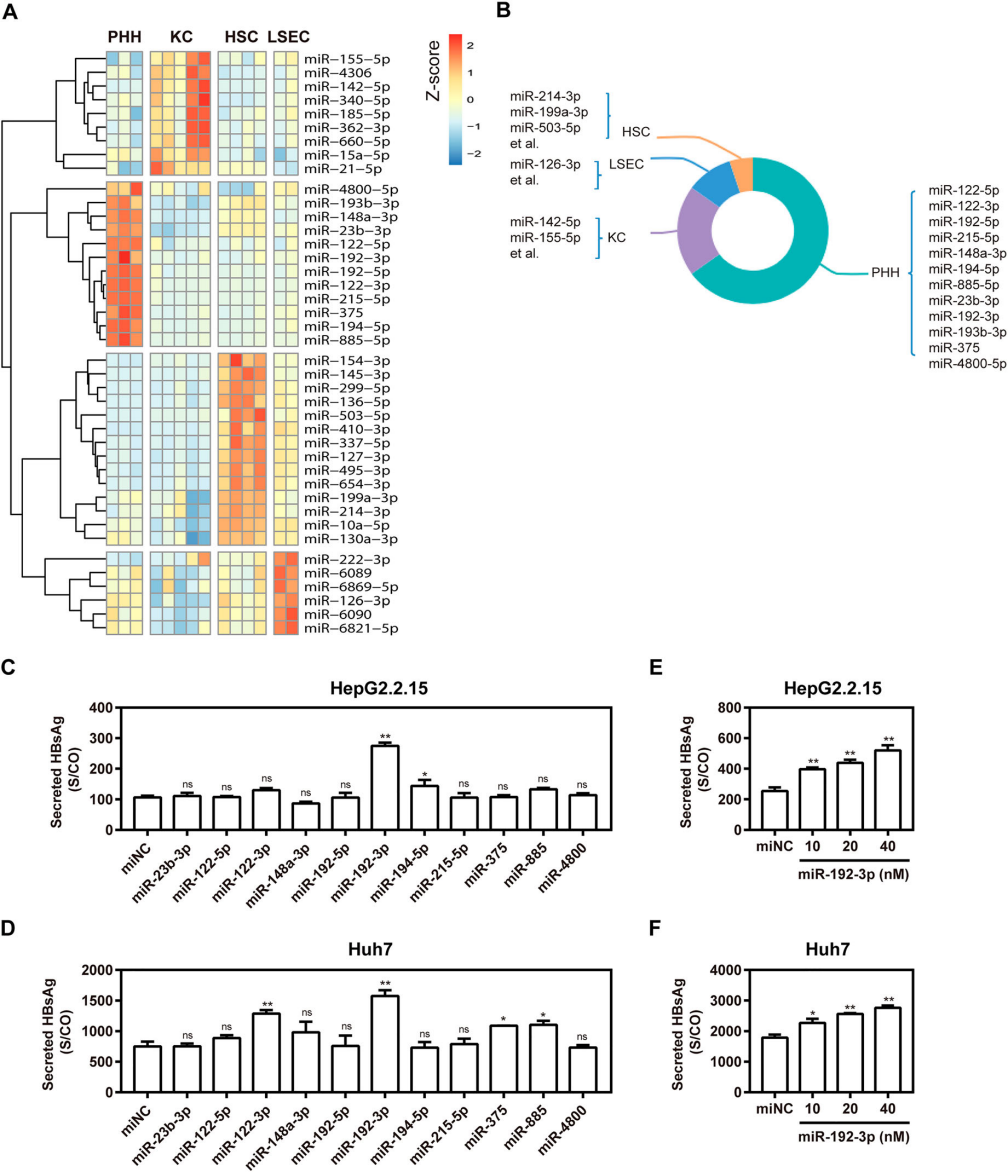
Enriched miRNA profiles for different hepatic cell types in human liver and effect of primary hepatocyte-enriched miRNAs on HBV production. (A) Heatmap of miRNAs with higher expression levels in PHH, KC, LSEC, or HSC, with a difference of two-fold or greater compared with other cell types. (B) Diagrammatic drawing of hepatic cell type-enriched miRNA profiles. (C) HepG2.2.15 cells were transfected with 11 specific miRNA mimics or negative control (miNC) at 40 nM; (D) Huh7 cells were co-transfected with plasmid pSM2 and different miRNA mimics or miNC at 40 nM, and harvested at 72 h post-transfection. Specific miR-192-3p mimics at different concentrations (0, 10, 20, and 40 nM) were separately transfected into HepG2.2.15 (E) and Huh7 (F) cells and harvested at 72 h post-transfection. Secreted HBsAg level in culture supernatants was determined by chemiluminescence immunoassay. *P < .05; **P < .01; ns, no significance. PHH, primary human hepatocyte; KC, Kupffer cell; LSEC, liver sinusoidal endothelial cell; HSC, hepatic stellate cell.

PHHs, LSECs, KCs, and HSCs, which represent the major fractions of hepatic cells, were prepared, and cell type-specific miRNA expression profiles were determined by miRNA microarray analysis. A total of 2549 human miRNAs were analysed according to miRBase edition 21.0. miRNAs detectable in more than 50% of the samples were defined as present in one cell type or liver tissue. Thus, 447, 437, 361, and 437 miRNAs were present in PHHs, KCs, HSCs, and LSECs, respectively (Table S4). miRNAs that were overexpressed more than two-fold were considered cell type-enriched. The hepatic cell type-enriched miRNA profiles are shown in a heat map (Figure 1(A)) and a pie graph (Figure 1(B)). Twelve miRNAs, including miR-122-5p, miR-122-3p, miR-192-3p, and others, were enriched in PHHs (Figure 1(A)). Among these, miR-122-5p and miR-122-3p showed the highest expression levels in PHHs compared with those in other cell types. Nine miRNAs, such as miR-142-5p and miR-155-5p, were enriched in KCs (Figure 1(A)). Among these, miR-142-5p and miR-155-5p were overexpressed at least four-fold in KCs compared with the other three cell types. Six miRNAs, including miR-126-3p and miR-222-3p, were enriched in LSECs (Figure 1(A)). Among these, miR-126-3p was expressed in LESCs more than four-fold than in the other three cell types. Fourteen enriched miRNAs were identified in HSCs (Figure 1(A)). Among these, miR-214-3p, miR-199a-3p, miR-10a-5p, and miR-130a-3p were enriched in the liver. The miR-214-3p and miR-199a-3p were overexpressed more than four-fold in HSCs.

### MiR-192-3p increases HBV replication and transcription level in hepatic cell lines

**Figure 2.**
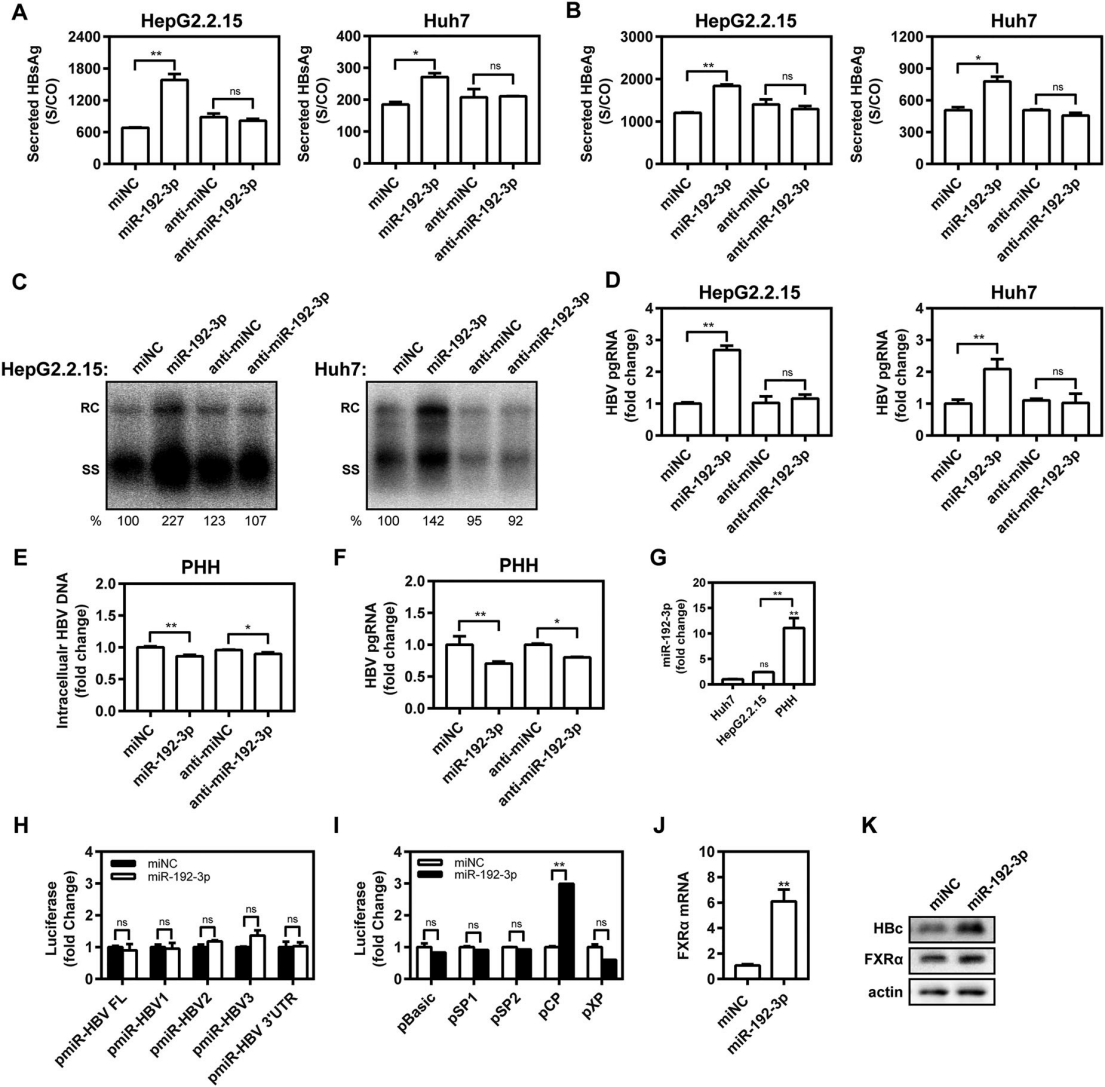
The miR-192-3p increases HBV replication and transcription level in hepatic cell lines. (A–D) HepG2.2.15 cells were transfected with miR-192-3p mimics or inhibitors at 40 nM; Huh7 cells were co-transfected with pSM2 plasmid and miR-192-3p mimics or inhibitors at 40 nM, and harvested at 72 h post-transfection. Secreted HBsAg (A) and (B) HBeAg levels in culture supernatants were determined by chemiluminescence immunoassay. (C) Encapsidated HBV replicative intermediates were isolated and detected by Southern blotting. (D) Intracellular HBV pgRNA level was determined by quantitative real-time RT-PCR analysis using primers matching to the pgRNA-specific region. (E, F) PHHs with HBV virion infection (MOI = 1000) were transfected with miR-192-3p mimics or inhibitors at 40 nM at 96 h post-HBV infection. At six days post-miRNA transfection, the intracellular encapsidated HBV replicative intermediates and pgRNA levels in PHHs were separately determined as described above. (G) The miR-192-3p levels in HepG2.2.15 and Huh7 cells as well as PHHs were measured by quantitative real-time RT-PCR analysis using specific primers. (H, I) Huh7 cells were co-transfected with miR-192-3p mimics or miNC at 40 nM and pMIR-REPORT plasmids (including pMIR-Luc, -HBV FL, -HBV1, -HBV2, -HBV3, or -HBV 3′UTR) or HBV promoter luciferase reporters containing the regions of pSP1, pSP2, pCP, and pXP) for 48 h with Renilla as an internal control. Dual-Glo luciferase report assay was performed to measure the firefly and Renilla luciferase activities. The results were calculated by fold change and normalized to the miNC samples. (J, K) HepG2.2.15 cells were transfected with miR-192-3p mimics or inhibitors at 40 nM and harvested at 72 h post-transfection. Nuclear receptor FXRα mRNA and protein levels were measured by quantitative real-time RT-PCR and Western blotting analysis, separately. *P < .05; **P < 0.01; ns, no significance. miNC, miRNA negative control; anti-miNC, miRNA inhibitor control; PHH, primary human hepatocyte; RC, relaxed circular DNA; SS, single-stranded DNA.

Eleven synthetic PHH-enriched miRNA mimics were transfected into HepG2.2.15 and Huh7 cells to test their effect on HBV production. Compared with miRNA negative control (miNC), miR-192b-3p strongly increased the supernatant HBsAg levels *in vitro* (Figure 1(C,D)), indicating the ability to facilitate HBV replication. Of note, miR-192-3p increased supernatant HBsAg level in a dose-dependent manner (Figure 1(E,F)). Moreover, we further found that miR-192-3p overexpression by transfecting the synthetic miR-192-3p mimics into the cells significantly increased the levels of secreted HBsAg and HBeAg in HepG2.2.15 and Huh 7 cells, but miR-192-3p inhibition showed no inhibitory effect on secreted HBsAg and HBeAg production (Figure 2(A,B)). Consistently, miR-192-3p significantly increased the amount of encapsidated HBV replicative intermediates (RIs) in HepG2.2.15 and Huh7 cells, but miR-192-3p inhibition showed no inhibitory effect on HBV DNA replication as well (Figure 2(C)). Moreover, the levels of intracellular HBV total (Figure S1) and pregenomic RNA (pgRNA; Figure 2(D)) were significantly increased by ectopic overexpression of miR-192-3p in HepG2.2.15 and Huh7 cells, but miR-192-3p inhibition showed no inhibitory effect on HBV transcription (Figure 2(D)). Interestingly, we observed that both miR-192-3p overexpression and inhibition caused the drop of intracellular HBV DNA (Figure 2(E)) and pgRNA levels (Figure 2(F)) in HBV-infected PHHs. To investigate the potential reason for HBV DNA drop caused by miR-192-3p overexpression, real-time RT-PCR was developed to measure the miR-192-3p levels in PHHs and hepatoma carcinoma cells. Our data showed that the miR-192-3p levels expressed in HepG2.2.15 and Huh7 cells were significantly much lower than that in PHHs (Figure 2(G)). The result implied that while anti-miR-192-3p reduced HBV RNA, additional miR-192-3p was toxic to the cells. Collectively, miR-192-3p overexpression increases HBV replication and transcription level in different hepatoma carcinoma cells.

### MiR-192-3p promotes HBV core promoter activity in Huh7 cells

To test whether miR-192-3p has a direct interaction with HBV genome or transcripts, pMIR-REPORT plasmids harbouring the full-length HBV genome or partial fragments and miR-192-3p mimics were co-transfected into Huh7 cells. We revealed that miR-192-3p did not interfere with the luciferase activities of different HBV fragments (Figure 2(H)). Thus, there was apparently no direct interaction between HBV genome or transcripts and miR-192-3p. Subsequently, the effect of miR-192-3p on the activity of four HBV promoters was further tested by using Dual-Glo luciferase report assays. The results showed that the luciferase activity of the HBV core promoter was significantly upregulated by miR-192-3p overexpression (Figure 2(I)), but without obvious effects on SP1, SP2, and X of HBV promoters. Further, we screened several HBV transcriptional factors and found that the transcriptional and expression level of bile acid nuclear receptor FXRα was significantly increased by miR-192-3p (Figure 2(J,K)). Expression of HBV core protein was also obviously increased after treating with miR-192-3p (Figure 2(K)). Taken together, these results suggest that miR-192-3p increases HBV replication and transcription level through upregulating nuclear receptor FXRα expression and promoting HBV core promoter activity in Huh7 cells.

### ZNF143 is a direct target of miR-192-3p

**Figure 3.**
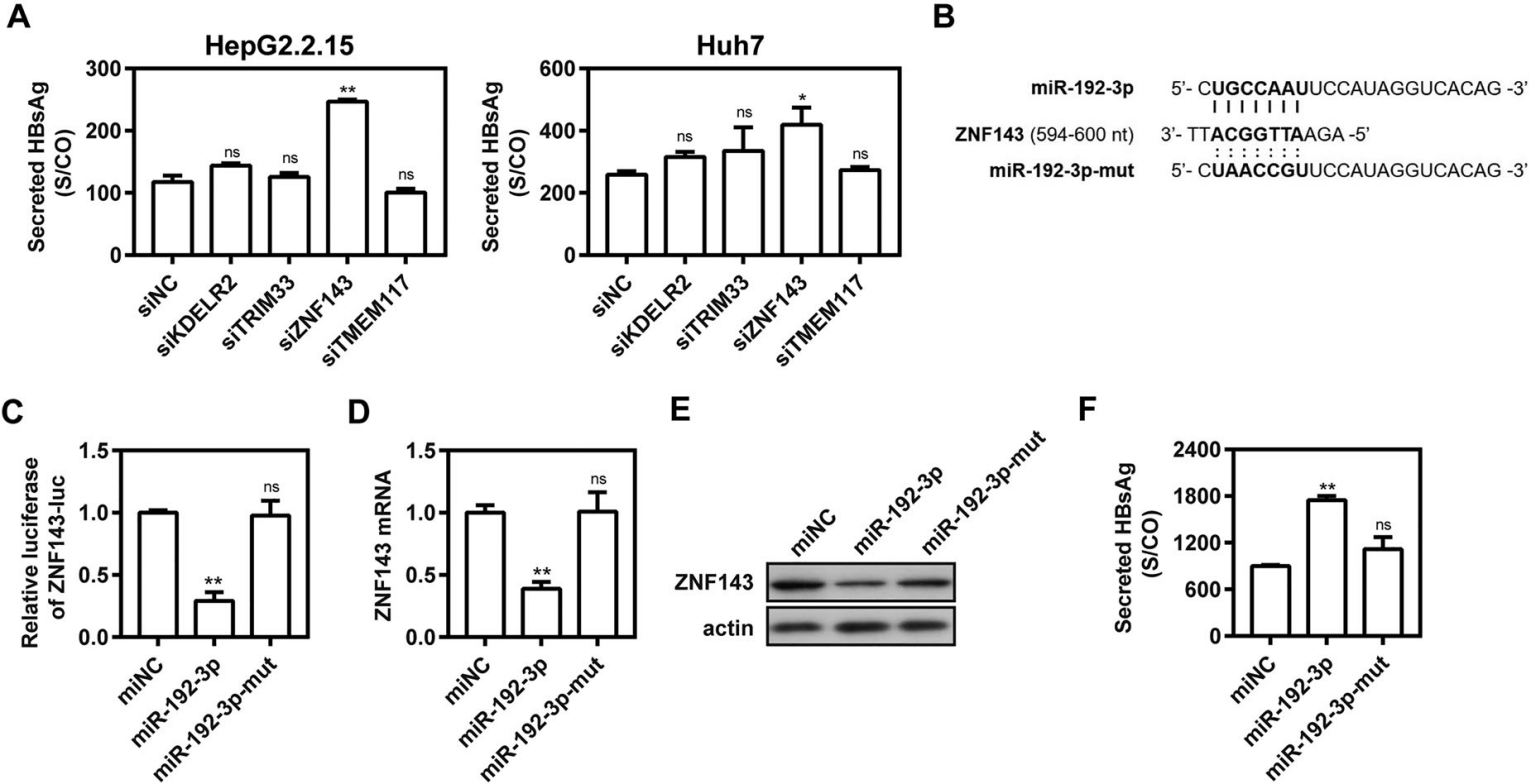
ZNF143 is a direct target of miR-192-3p. (A) HepG2.2.15 cells were transfected with different specific siRNAs or negative control (siNC) at 40 nM; Huh7 cells were co-transfected with pSM2 plasmid and different siRNAs or siNC at 40 nM, and harvested at 72 h post-transfection. Secreted HBsAg level in culture supernatants was determined by chemiluminescence immunoassay. (B) Schematic diagram of miR-192-3p-binding sites within the consequences of ZNF143 mRNA 3′UTR. (C) HepG2.2.15 cells were co-transfected with miR-192-3p mimics or miNC at 40 nM and ZNF143 luciferase reporter plasmid containing its 3′UTR nucleotide consequences with Renilla as an internal control. The data from dual-Glo luciferase report assay were calculated by fold change and normalized to the miNC samples. (D–F) HepG2.2.15 cells were transfected with miR-192-3p mimics or inhibitors at 40 nM and harvested at 72 h post-transfection. ZNF143 mRNA (D) and protein (E) levels were measured by quantitative real-time RT-PCR and Western blotting analysis, separately. (F) Secreted HBsAg level in culture supernatants was determined as described above. *P < .05; **P < .01; ns, no significance.

TargetScan (http://www.targetscan.org/vert_72/) was used for computational prediction analysis and identified several potential target genes of miR-192-3p, such as KDEL endoplasmic reticulum protein retention receptor 2 (KDELR2), tripartite motif containing 33 (TRIM33), transmembrane protein 117 (TMEM117), and Zinc finger protein 143 (ZNF143). To date, there have been no reports as to whether these genes participate in HBV replication. Knocking down these four genes with specific siRNAs in HepG2.2.15 and Huh7 cells showed that silencing of ZNF143 significantly increased supernatant HBsAg level, indicating that ZNF143 gene may be involved in viral replication (Figure 3(A)). As shown in Figure 3(B), miR-192-3p-binding sites were found within the 3′-UTR of ZNF143 mRNA. The Dual-Glo luciferase reporter assay showed that miR-192-3p can suppress luciferase expression of ZNF143 gene (Figure 3(C)). Consistently, transfection of miR-192-3p significantly decreased ZNF143 mRNA (Figure 3(D)) and protein expression levels (Figure 3(E)). MiR-192-3p expression increased the levels of secreted HBsAg and HBeAg, while the miR-192-3p mutation in seed sequence abolished the promoting effect (Figure 3(F)). Therefore, ZNF143 is a direct target of miR-192-3p to modulate HBV replication in hepatic cell lines.

### ZNF143 modulates HBV replication through regulating HBV core promoter activity in hepatic cell lines

**Figure 4.**
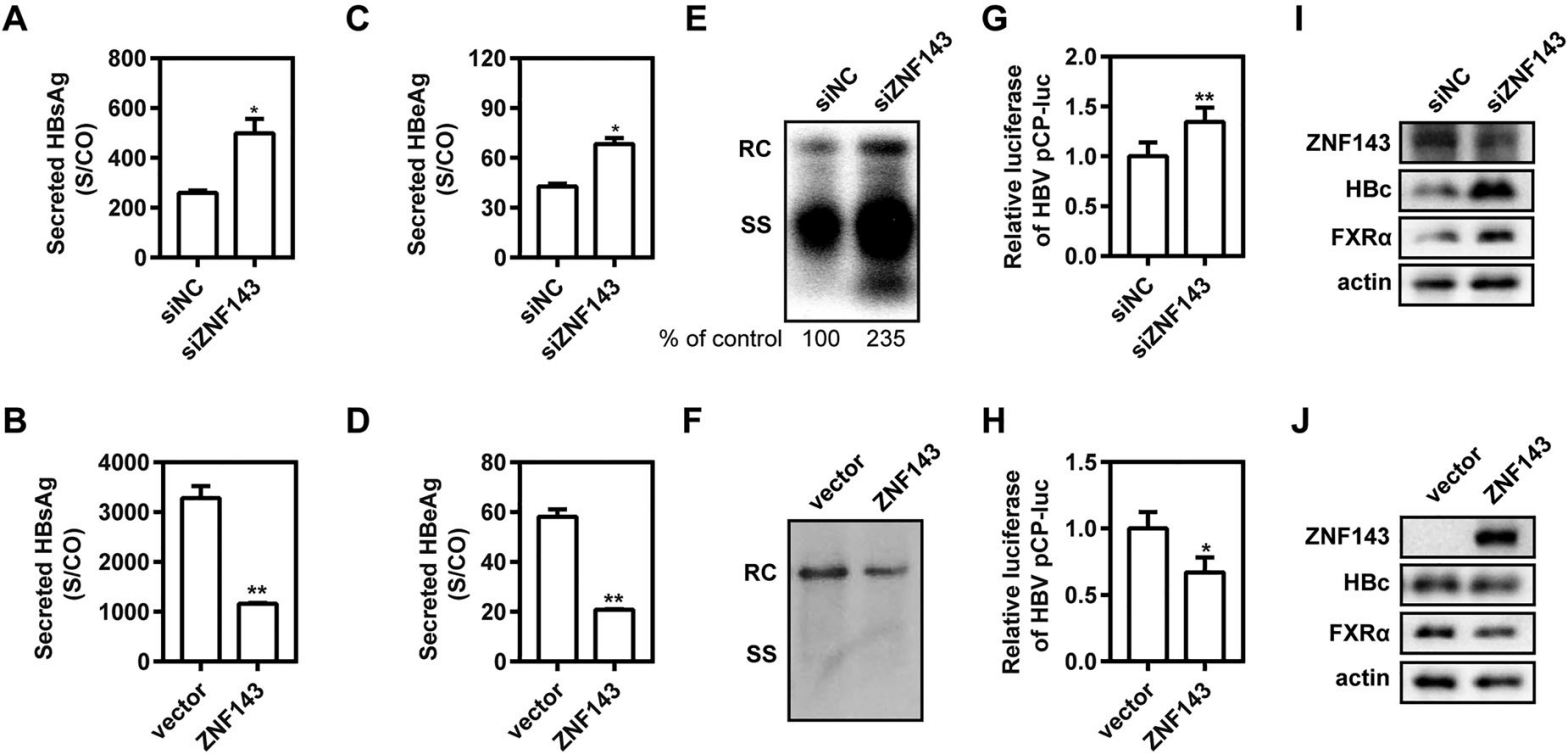
ZNF143 modulates HBV replication through regulating HBV core promoter activity in hepatic cell lines. (A–F) Huh7 cells were co-transfected with plasmid pSM2 and specific siRNAs against ZNF143 (siZNF143) at 40 nM (A, C, E) or plasmid Flag-ZNF143 (B, D, F), and harvested at 72 h post-transfection. Secreted HBsAg (A, B) and HBeAg (C, D) levels in culture supernatants were determined by chemiluminescence immunoassay. (E, F) Encapsidated HBV replicative intermediates were isolated and detected by Southern blotting. (G–J) Huh7 cells were co-transfected with siZNF143 or negative control (siNC) at 40 nM and HBV promoter luciferase reporters containing the region of pCP for 48 h with Renilla as an internal control. The data from dual-Glo luciferase report assay were calculated by fold change and normalized to the siNC samples. HepG2.2.15 (I) and Huh7 cells (J) were separately treated as (A–F). At 72 h post-transfection, western blotting analysis was performed to detect the levels of ZNF143, FXRα, and HBV core proteins using beta-actin as a loading control. *P < .05; **P < .01; ns, no significance. RC, relaxed circular DNA; SS, single-stranded DNA.

ZNF143 is a transcription activator regulating cell cycle-associated genes, but there are rare reports as to whether it is involved in virus replication [21]. To further explore the effect of ZNF143 on HBV replication, ZNF143 silencing or overexpression was performed in different hepatoma cell lines. We found that ZNF143 silencing by transfection of specific siRNAs against ZNF143 into Huh7 cells increased the levels of secreted HBsAg (Figure 4(A)) and HBeAg (Figure 4(C)) and enhanced HBV RIs (Figure 4(E)), whereas ZNF143 overexpression decreased the levels of secreted HBsAg (Figure 4(B)) and HBeAg (Figure 4(D)) and reduced HBV RIs (Figure 4(F)). Similar results were observed in HepG2.2.15 cells (Figure S2). Moreover, ZNF143 silencing significantly increased the activity of HBV core promoter (Figure 4(G)). In contrast, ZNF143 overexpression significantly decreased the activity of HBV core promoter (Figure 4(H)). Additionally, we further observed that ZNF143 silencing significantly enhanced the levels of HBV core protein and nuclear receptor FXRα expression (Figure 4(I)). Consistently, ZNF143 overexpression decreased HBV core protein level and nuclear receptor FXRα expression (Figure 4(J)). These results indicate that ZNF143 modulates HBV replication through regulating HBV core promoter activity in hepatic cell lines.

### MiR-192-3p increases HBV replication through inhibiting ZNF143/Akt/mTOR signalling in HepG2.2.15 cells

**Figure 5.**
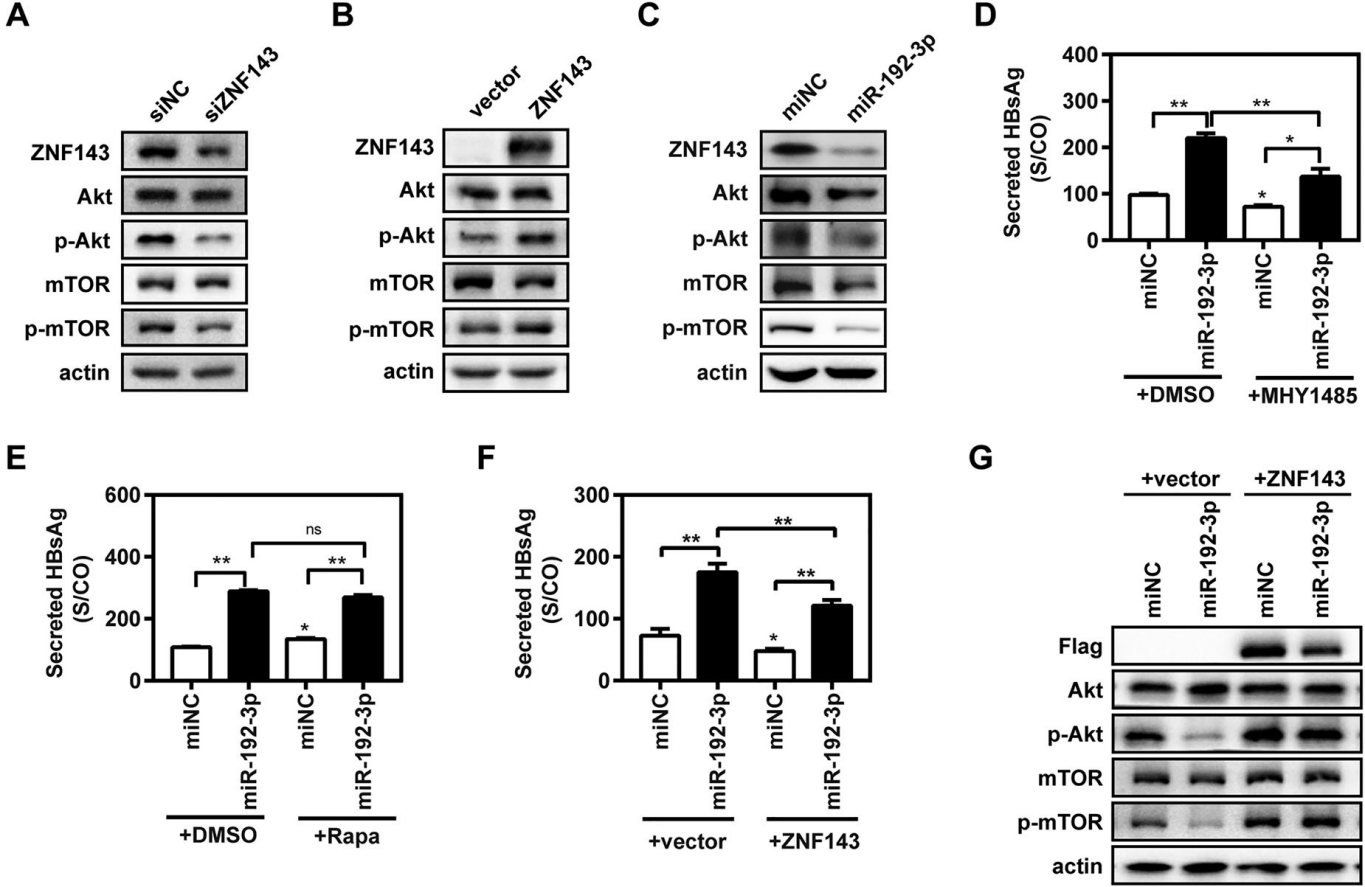
MiR-192-3p increases HBV replication through inhibiting ZNF143/Akt/mTOR signalling in HepG2.2.15 cells. (A–C) HepG2.2.15 cells were transfected with specific siRNAs against ZNF143 (siZNF143) or negative control (siNC) at 40 nM; plasmid Flag-ZNF143 or empty vector pCMV-10; miR-192-3p mimics or negative control (miNC) at 40 nM, and harvested at 72 h post-transfection. Total or phosphorylated Akt and mTOR protein levels were measured by western blotting. HepG2.2.15 cells were transfected with specific miR-192-3p mimics or miNC at 40 nM. At 24 h post-miRNA transfection, the cells were treated with 2 µM MHY 1485 (D) or Rapamycin (D) for 48 h. (E, F) HepG2.2.15 cells were co-transfected with miR-192-3p mimics at 40 nM and plasmid Flag-ZNF143 or empty vector pCMV-10 and harvested at 72 h post-transfection. Secreted HBsAg level in culture supernatants was determined by chemiluminescence immunoassay. Total or phosphorylated Akt and mTOR protein levels were analysed by Western blotting. *P < .05; **P < .01; ns, no significance.

It was previously reported that ZNF143 enhanced metastasis of gastric cancer cells by promoting the process of epithelial to mesenchymal transition through the PI3K/Akt signalling pathway [21]. Meanwhile, other studies revealed that the inhibition of the Akt/mTOR signalling could facilitate HBV replication by enhancing autophagy [16,22]. It is still unknown whether ZNF143 regulates HBV replication through modulating the Akt/mTOR signalling pathway. ZNF143 silencing obviously reduced the levels of ZNF143, phosphorylated Akt (p-Akt), and p-mTOR expression (Figure 5(A)) in HepG2.2.15 cells, whereas ZNF143 overexpression increased the levels of p-Akt and p-mTOR expression (Figure 5(B)). MiR-192-3p overexpression also significantly decreased the levels of ZNF143, p-Akt, and p-mTOR (Figure 5(C)). Our data revealed that the mTOR activator MHY1485 significantly, but not completely, impaired the promoting effect of miR-192-3p on secreted HBsAg (Figure 5(D)) and HBeAg (Figure S3(A)) levels in HepG2.2.15 cells. Secreted HBsAg (Figure 5(E)) and HBeAg (Figure S3(B)) levels in supernatants were significantly increased in the Rapamycin group than that in the control DMSO group, whereas Rapamycin had a much weaker promoting effect on viral replication than miR-192-3p overexpression did. Moreover, ZNF143 overexpression partly impaired the enhancement effect of miR-192-3p on secreted HBsAg production in Huh7 cells. (Figure 5(F)). Western blot result further showed that ZNF143 overexpression can offset the inhibitory effect of miR-192-3p on the Akt/mTOR signalling pathway (Figure 5(G)). Collectively, these results indicate that miR-192-3p enhances HBV replication through inhibiting Akt/mTOR signalling by targeting ZNF143 in HepG2.2.15 cells.

### Circulating and hepatic miR-192-3p expression are increased in CHB patients

**Figure 6.**
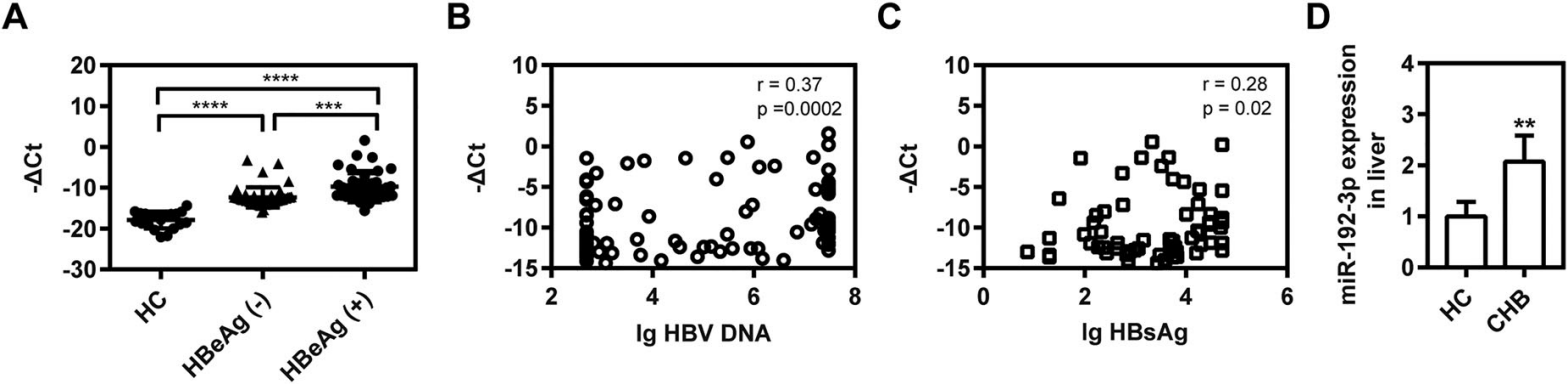
Circulating and hepatic miR-192-3p expression are increased in CHB patients. (A) Distribution of serum miR-192-3p levels in HBeAg-positive or negative patients and healthy controls. Serum miR-192-3p level was measured by quantitative real-time RT-PCR analysis using specific primers. Correlation analyses of serum miR-192-3p levels and HBV DNA loads (B) or HBsAg levels (C) in CHB patients. (D) The hepatic miR-192-3p levels in CHB and healthy controls. *P < .05; **P < .01; ns, no significance.

The level of serum miR-192-3p was measured in 109 CHB patients by real-time qRT-PCR. Our data revealed that HBeAg-positive CHB patients had a higher level of serum miR-192-3p than HBeAg-negative patients and HBV-infected patients had a higher level of serum miR-192-3p than healthy controls (Figure 6(A)). Moreover, the levels of serum miR-192-3p positively correlated with serum HBV DNA and HBsAg levels (Figure 6(B,C)), which were consistent with the results *in vitro*. Thus, the circulating miR-192-3p positively correlates with serum HBV DNA and HBsAg levels in CHB patients. Moreover, we further detected the levels of hepatic miR-192-3p expression in CHB patients and healthy controls using real-time RT-PCR. Our data showed that CHB patients had higher hepatic miR-192-3p levels than healthy controls (Figure 6(D)), indicating that there is a positive correlation of miR-192-3p level with HBV DNA load. Collectively, the levels of circulating and hepatic miR-192-3p expression are increased in CHB patients.

## Discussion

A variety of physiological and pathological biological processes are regulated by miRNAs. Most miRNAs are relatively conserved in the evolutionary progress [8]. In the recent Functional Annotation of the Mammalian Genome project, cell-type specificity of miRNA expression was calculated across all collections of primary cell types, showing that almost half of the expressed miRNAs are cell type-enriched [23]. Thus, it is fundamental to characterize the expression profiles of miRNAs across cell types and tissues to understand and study their roles in the suitable cellular context. In this study, we identified the miRNA profiles enriched in four hepatic cell types, including PHH, KC, LSEC, and HSC. Further, the role of hepatic-enriched miRNA in the regulation of viral replication and the mechanism of PHH-enriched miR-192-3p in the regulation of viral replication were investigated.

It is well known that miR-122-5p is specific to PHHs [8]. Another study showed that miR-126-3p was specific to LSECs in rats [10]. Interestingly, the levels of most PHH-enriched miRNAs identified in this study (including miR-122-5p/3p, miR-192-3p, miR-215-5p, miR-148a-3p, miR-194-5p, miR-885-5p, and miR-193b-3p) were reported to be significantly lower in hepatocellular carcinoma tissue than those in normal liver tissue (Figure S4) [24]. A recent study also reported that intrahepatic miR-885-5p, miR-122-3p, miR-375, miR-194-5p, miR-192-5p, miR-193b-3p, and miR-148a-3p were most dramatically reduced in patients with HBV-associated acute liver failure [25]. Our study found that the miR-192-3p level was much lower in hepatoma carcinoma cells than that in PHHs (Figure 2(G)). Moreover, the clinical data further revealed that hepatic miR-192-3p levels were significantly higher in CHB patients than those in healthy controls (Figure 6(D)), indicating that there is a positive correlation of miR-192-3p level with HBV DNA load. Additionally, miR-192-3p overexpression inhibited the proliferation of hepatoma carcinoma cells by targeting p-Rb (Figure S5). Collectively, these studies have indicated that these PHH-enriched miRNAs are substantial to maintain regular proliferation and differentiation of hepatocytes and that their dysregulation causes liver pathogenesis.

Many miRNAs were reported to be involved in the HBV life cycle. Our previous studies reported that miR-1, miR-125b, and miR-99 family could facilitate viral replication [16–18]. In the current study, several PHH-enriched miRNAs were observed to facilitate HBV replication at different levels, and miR-192-3p showed the greatest pro-virus effect in hepatic cell lines. Further, serum miR-192-3p showed a positive correlation with HBV DNA and HBsAg, which was consistent with the previous study [26,27]. Meanwhile, higher hepatic miR-192-3p levels were observed in CHB patients than in healthy controls. Previously, miR-192-3p was reported to directly target cell cycle-promoted factor CCNB1 and suppress non-small cell lung cancer cell growth [28]. Hepatic miR-192-3p reactivation alleviated steatosis by targeting glucocorticoid receptor [29]. Recently, Wang et al. reported that miR-192-3p targeted X-linked inhibitor of apoptosis protein to inhibit autophagy through the nuclear factor kappa β signalling pathway to reduce HBV replication [30]. In our present study, we revealed that serum miR-192-3p level was positively correlated with serum HBV DNA load and HBsAg level, as well as promoting HBV transcription/replication. Winther et al. has reported that serum miR-192-3p level was significantly higher in HBeAg-positive patients than HBeAg-negative patients [26]. Moreover, serum miR-192-3p level in CHB patients of immune tolerant phase was significantly higher than that in immune control phase [31]. Additionally, its homology miR-192-5p level in HBeAg-positive patients was also significantly higher than that in HBeAg-negative patients, as well as being positively correlated with serum HBV DNA load and HBsAg level [26,27,31,32]. However, the positive correlation between serum miR-192-3p level and serum HBsAg/viral load from these published papers and our present study was only contradictory to Wang’s findings [30]. Collectively, our present findings were in accordance with the results of all published works only except Wang’s conclusion. As to the possible cause, it remains unclear for us and further exploration may be warranted.

In our present study, the miR-192-3p enhanced HBV replication and transcription in Huh7 and HepG2.2.15 cells. However, we observed that miR-192-3p overexpression and inhibition both downregulated the intracellular HBV DNA and pgRNA levels in HBV-infected PHHs (Figure 2(E,F)). As a class of noncoding RNAs, miRNA may have various functions by targeting the 3′UTR of potential gene mRNA. Our previous studies have found that some tumour-suppressive miRNAs, including miR-1 [18], miR-99a/b, miR-100 [16], miR-125b [17], and miR-192-3p, not only have the similar ability on promoting HBV replication but also modulating cell proliferation and apoptosis. In accordance with the inhibitory effect of miR-192-3p overexpression in PHHs in this study, we previously also observed that ectopic miR-1 overexpression caused HBV RNA/DNA drop in PHHs [18]. Additionally, Li et al. revealed that ectopic miR-1 overexpression also induced apoptosis of hepatoma HepG2 cells by targeting API-5 [33], indicating that there would be an inner balance between cell survival (conducive for HBV replication) and apoptosis (bad for HBV replication). From our real-time RT-PCR results, we observed that there was much higher miR-192-3p level in PHHs than that in hepatoma HepG2.2.15 and Huh7 cells (Figure 2(G)). Therefore, ectopic miR-192-3p overexpression may cause more severe apoptosis and lead to reduced HBV replication in miR-192-3p-enriched PHHs. The facilitating effect of miR-192-3p overexpression on HBV replication is applied to hepatoma cell lines with its low expression (such as HepG2.2.15 and Huh7 cells), but not to PHHs with its high expression.

Furthermore, it was observed that miR-192-3p overexpression increased HBV replication and transcription level through inhibiting Akt/mTOR signalling by targeting ZNF143 in HepG2.2.15 cells. MiR-192-3p upregulated nuclear receptor FXRα expression and promoted HBV core promoter activity. Guo et al. confirmed that activation of the PI3K/Akt pathway profoundly suppresses the transcription of HBV pgRNA and 2.4-kb mRNA, but not 2.1-kb mRNA, indicating that suppressing mTOR to activate core and pre-S1 promoter [34]. Teng et al. also demonstrated the mechanism of activation of mTOR in suppressing pre-S1 and was a further explanation of Guo’s discovery [22]. MTORC1 is one of the critical intermediate regulator molecules controlling regulating cell growth, autophagy, cellular metabolism, etc., which is regulated by growth factors (such as insulin and other growth factors) or nutrient stimulators [35,36]. In our previous study, miR-99 family inhibited the mTORC1 signalling increased HBV replication without significantly increasing pgRNA level [16]. We further revealed that miR-99 family promoted viral replication through mTOR/ULK1 signalling pathway-induced autophagy, which was post-transcriptionally upregulated HBV replication [16]. However, Guo’s and our present studies supported that HBV replication was transcriptionally upregulated by mTORC1 inhibition without elucidating the definite molecular mechanism. In our findings, the enhanced HBV replication induced by miR-192-3p overexpression was closely related to the upregulated nuclear factor FXRa expression and HBV core promoter activity. We noticed that the treatment with mTOR activator cannot completely block the enhancement effect of miR-192-3p on HBV replication. This indicates that there would be other potential mechanisms of modulating HBV replication by unverified targets of miR-192-3p.

ZNF143 is a pervasive transcriptional activator regulating many cell cycle-associated genes [37]. The human ZNF143 gene encodes a 638 amino acid transcriptional activator protein consisting of a well-characterized tandem zinc finger DNA-binding domain and two separable activation domains [38]. ZNF143 is a positive regulator of many cell cycle genes as well as a potential co-factor involved in chromatin looping and establishing a higher-order structure within the genome [39,40]. It has been reported that ZNF143-mediated H3K9 trimethylation upregulated CDC6 in hepatocellular carcinoma [41]. ZNF143 was also observed to enhance metastasis of gastric cancer by promoting the process of epithelial to mesenchymal transition through the PI3K/AKT signalling pathway [21]. Previously, no reports confirmed that ZNF143 could regulate HBV replication, and our study was the first to identify that ZNF143 is able to suppress HBV replication. Inhibition of the Akt/mTOR signalling pathway was reported to enhance HBV replication [16,22]. Based on the previous study, we observed that miR-192-3p targets ZNF143 and downregulation of ZNF143 caused inhibition of Akt/mTOR signalling, elucidating the mechanism of PHH-enriched miR-192-3p in the facilitation of HBV replication. To test whether ZNF143 has a direct effect on HBV core promoter, TF-binding motif sequences of ZNF143 were predicted as TACCCACAATGCATTG (homo sapiens) with the aid of JASPAR database. We found that there was no putative binding site in the whole sequences of HBV core promoter, indicating that ZNF143 may not have a direct effect on HBV core promoter. Subsequently, we further scanned the sequences of some presentative nuclear factors (including PPARα, HNF4α, and FXRα), which were known to directly regulate HBV core promoter activity. Three putative binding sites in HNF4a and one in FXRα were predicted with TF-binding motif sequences of ZNF143. Subsequently, we further investigated the effect of ZNF143 overexpression on HNF4a and FXRα mRNA levels. We found that only FXRα protein expression was significantly downregulated by ZNF143 overexpression, whereas upregulated by ZNF143 silencing. Therefore, we propose that ZNF143 may promote HBV core promoter activity by upregulating FXRα expression. A detailed mechanism needs further investigation in the future.

MiRNAs exist stably in circulation for packaging into lipid vesicles or glycoproteins. Nearly 90% of circulating miRNAs exist as complexes with specific proteins like Ago, HDL, or other RNA-binding proteins and the remaining include exosomes or microparticles [42]. Serum miRNA closely correlates with intrahepatic miRNA and reflects the miRNA profile in the liver to some extent [43]. All PHH-specific miRNAs identified were previously reported or determined in our study to show higher serum levels in HBV-infected patients than in healthy people, and were higher in HBeAg positive than in HBeAg-negative patients [27,44,45]. In our study, serum miR-192-3p positively correlated with HBV DNA load and HBsAg levels. As previously reported, circulating HBsAg particles also carry miRNAs such as miR-122, miR-223, and miR-145 in CHB patients [46]. Till now, it is not clear if the higher levels of miR-192-3p in patients with higher viral load and biochemical activity is due to a real overexpression in hepatic tissue or it is the result of a higher release in serum because of the cell lysis. Therefore, we detected the level of hepatic miR-192-3p expression from CHB patients and normal liver tissue from resection of hepatic haemangioma. The result shows that CHB patients had higher hepatic miR-192-3p levels than healthy controls, indicating higher serum levels of miR-192-3p in CHB patients is probably due to a real overexpression in hepatic tissue. miR-155-5p, the most abundant in lymphocytes, trended to be higher in the immune active phase compared to the immune tolerate phase which was probably caused by numerous HBV-specific T cells infiltrating the liver [23]. HSC-specific miR-214-3p in the liver reflected the severity of liver fibrosis [47]. Therefore, hepatic cell type-specific miRNAs may serve as potential biomarkers for HBV-related liver diseases.

In conclusion, our study determined the four kinds of hepatic cell type-enriched miRNA expression profiles. The effect of PHH-enriched miRNAs on HBV replication was tested and the mechanism of PHH-enriched miR-192-3p in the regulation of viral replication was elucidated. Our findings provide a specific perspective for the role of hepatic cell type-enriched miRNAs in viral replication and various liver pathogenesis.

## Supplementary Material

Supplemental Material

Supplemental Material
