# Supplementary material for: Human hepatocyte-enriched miRNA-192-3p promotes HBV replication through inhibiting Akt/mTOR signalling by targeting ZNF143 in hepatic cell lines
Source: Emerg Microbes Infect. 2022 Feb 21;11(1):616–28. doi: 10.1080/22221751.2022.2037393 (PMC8865105; doi:10.1080/22221751.2022.2037393)
Supplement: Supplemental Material [file TEMI_A_2037393_SM2033.docx]

| ID | Gender | Age | ALT (U/L) | AST (U/L) | GGT  (U/L) | HBsAg | Anti-HCV | Indication for resection | Type of surgery |
| --- | --- | --- | --- | --- | --- | --- | --- | --- | --- |
| Pat. 1 | m | 76 | 122 | 181 | 20 | negative | negative | Colon Cancer, liver metastasis | Resection |
| Pat. 2 | m | 59 | 34 | 72 | 52 | negative | negative | HCC | Resection |
| Pat. 3 | m | 67 | 19 | 23 | 26 | negative | negative | Colon Cancer, liver metastasis | Resection |
| Pat. 4 | m | 64 | 27 | 34 | 81 | negative | negative | Colon Cancer, liver metastasis | Resection |
| Pat. 5 | m | 78 | 37 | 31 | 25 | negative | negative | Colon Cancer, liver metastasis | Resection |
| Pat. 6 | m | 68 | 46 | 36 | 222 | negative | negative | Caroli disease | Resection |
| Pat. 7 | f | 51 | 21 | 19 | 194 | negative | negative | Colon Cancer, liver metastasis | Resection |

**Supplementary Table S1. The** **demographic features of patients for isolation of hepatic cell types**

Abbreviations: HCC, hepatocellular cell carcinoma; m, male, f, female; pat, patient.

**Supplementary Table S2. Clinical and virological features of Chronic Hepatitis B patients enrolled for serum miRNA detection**

|  | HBeAg positive patients  (n=52) | HBeAg-negative patient  (n=57) | Healthy control  （HC, n=20） |
| --- | --- | --- | --- |
| Age (years)* | 30 (27-34) | 37.5 (30.25-47) | 32 (24-52) |
| Gender (M/F) | 23/29 | 38/19 | 14/6 |
| HBV DNA  (log_10_ IU/mL) * | 7.34 (5.91-7.47) | 2.69 (2.69-2.69) | NA |
| HBsAg  (log_10_ IU/mL)* | 4.27 (3.68-4.68) | 2.64 (1.95-3.43) | NA |
| HBeAg(+/-) | 52/0 | 0/57 | NA |
| ALT (U/L)* | 47(27-105) | 42 (21.5-70) | 23 (7-40) |
| AST (U/L)* | 35(21.75-64) | 27.5(19.25-43.75) | 24 (13-35) |

Abbreviations: Healthy control, HC; M, male; F, female.* expressed as median (25% percentile – 75% percentile).

**Supplementary Table S3. Primers used for real time PCR and luciferase reporter cloning**

| **Gene name** | **Application** | **Type** | **Sequence 5’-3’** | **Position of 5’-base** |
| --- | --- | --- | --- | --- |
| HBV DNA | real time PCR | forward | GTTGCCCGTTTGTCCTCTAATTC | 465 |
|  |  | reverse | GGAGGGATACATAGAGGTTCCTT | 563 |
| HBV total RNA | real time RT-PCR | forward | CCGTCTGTGCCTTCTCATCTGC | 1551 |
|  |  | reverse | ACCAATTTATGCCTACAGCCTCC | 1800 |
| HBV pgRNA | real time RT-PCR | forward | CTGGGTGGGTGTTAATTTGG | 2112 |
|  |  | reverse | TAAGCTGGAGGAGTGCGAAT | 2297 |
| SP1 promoter | pSP1 cloning | forward | cgacgcgt CTCACTTTTGGAAGAGAAAC | 2224 |
|  |  | reverse | CCTAGATCT CTTATATAATATACCCGC | 2784 |
| SP2 promoter | pSP2 cloning | forward | cgacgcgt GTGGGTCACCATATTCTTG | 2814 |
|  |  | reverse | CCTAGATCT CTTCCTGACTGGCGATTG | 3123 |
| Core promoter | pCP cloning | forward | cgacgcgt GGTCTTACATAAGAGGAC | 1648 |
|  |  | reverse | CCTAGATCT TGAACAAGAGATGATTAG | 1853 |
| X promoter | pXP cloning | forward | cgacgcgt TGCGTGGAACCTTTTCGG | 1237 |
|  |  | reverse | CCTAGATCT TGGAAACGATGTATATTTG | 1375 |
| HBV1 | pmiR-HBV1 | forward | cgacgcgt GATCTACAGCATGGGGCAGA | 2840 |
|  |  | reverse | cccaagctt TTTAAATGTATACCCAAAGAC | 837 |
| HBV2 | pmiR-HBV2 | forward | CGACGCGT AACCCTAACAAAACAAAGAG | 837 |
|  |  | reverse | CCCaagctt ATTAGGCAGAGGTGAAAAAG | 1840 |
| HBV3 | pmiR-HBV3 | forward | cgacgcgt CCTCTGCCTAATCATCTC | 1830 |
|  |  | reverse | cccaagctt TGCTGTAGATCTTGTTCCC | 2849 |
| HBV 3UTR | pmiR-HBV3UTR | forward | cgacgcgt TCATCTCTTGTTCATGTCCT | 1841 |
|  |  | reverse | CCCAAGCTT AGAAGGCAAAAACGAGAGTAA | 1964 |

The underlined parts of primers indicate the specific cleavage sites of restriction enzymes (“_”,MluI ; “_ _”, BglII; “….”HindIII).

Genebank accession No.: HBV, V01460.

**Supplementary table S4. MiRNA profile for each hepatic cell type**

| PHH | | KC | | HSC | | LSEC | | |  |
| --- | --- | --- | --- | --- | --- | --- | --- | --- | --- |
| miRNAs | Normalized signal value | miRNAs | Normalized signal value | miRNAs | Normalized signal value | | miRNAs | Normalized signal value | |
| hsa-let-7a-5p | 11.3 | hsa-let-7a-5p | 11.1 | hsa-let-7a-5p | 11.4 | | hsa-let-7a-5p | 11.0 | |
| hsa-let-7b-3p | 3.3 | hsa-let-7b-3p | 2.9 | hsa-let-7b-3p | 3.0 | | hsa-let-7b-3p | 3.8 | |
| hsa-let-7b-5p | 10.2 | hsa-let-7b-5p | 9.7 | hsa-let-7b-5p | 10.8 | | hsa-let-7b-5p | 10.6 | |
| hsa-let-7c-5p | 9.2 | hsa-let-7c-5p | 7.1 | hsa-let-7c-5p | 9.1 | | hsa-let-7c-5p | 9.1 | |
| hsa-let-7d-5p | 7.9 | hsa-let-7d-5p | 8.3 | hsa-let-7d-5p | 8.8 | | hsa-let-7d-5p | 8.2 | |
| hsa-let-7e-5p | 6.2 | hsa-let-7e-5p | 8.8 | hsa-let-7e-5p | 9.1 | | hsa-let-7e-5p | 8.2 | |
| hsa-let-7f-1-3p | 3.2 | hsa-let-7f-1-3p | 2.9 | hsa-let-7f-1-3p | 3.1 | | hsa-let-7f-1-3p | 4.0 | |
| hsa-let-7f-5p | 10.6 | hsa-let-7f-5p | 10.7 | hsa-let-7f-5p | 10.5 | | hsa-let-7f-5p | 10.2 | |
| hsa-let-7g-5p | 9.5 | hsa-let-7g-5p | 9.6 | hsa-let-7g-5p | 8.9 | | hsa-let-7g-5p | 8.2 | |
| hsa-let-7i-5p | 8.2 | hsa-let-7i-5p | 9.5 | hsa-let-7i-5p | 9.2 | | hsa-let-7i-5p | 9.3 | |
| hsa-miR-100-5p | 7.2 | hsa-miR-100-5p | 7.0 | hsa-miR-100-5p | 8.5 | | hsa-miR-100-5p | 9.2 | |
| hsa-miR-101-3p | 5.5 | hsa-miR-101-3p | 4.4 | hsa-miR-101-3p | 3.6 | | hsa-miR-101-3p | 3.3 | |
| hsa-miR-103a-3p | 8.2 | hsa-miR-103a-3p | 8.4 | hsa-miR-103a-3p | 8.3 | | hsa-miR-103a-3p | 9.0 | |
| hsa-miR-106b-5p | 5.6 | hsa-miR-106b-5p | 5.5 | hsa-miR-106b-5p | 5.9 | | hsa-miR-106b-5p | 6.4 | |
| hsa-miR-107 | 8.2 | hsa-miR-107 | 8.0 | hsa-miR-107 | 7.8 | | hsa-miR-107 | 7.9 | |
| hsa-miR-10a-5p | 4.9 | hsa-miR-10a-5p | 3.8 | hsa-miR-10a-5p | 7.6 | | hsa-miR-10a-5p | 6.4 | |
| hsa-miR-1202 | 5.4 | hsa-miR-1202 | 7.5 | hsa-miR-1202 | 5.9 | | hsa-miR-1202 | 7.3 | |
| hsa-miR-1207-5p | 5.2 | hsa-miR-1207-5p | 4.3 | hsa-miR-1207-5p | 3.4 | | hsa-miR-1207-5p | 5.7 | |
| hsa-miR-1225-5p | 5.4 | hsa-miR-1225-5p | 5.3 | hsa-miR-1225-5p | 5.0 | | hsa-miR-1225-5p | 6.3 | |
| hsa-miR-122-3p | 9.4 | hsa-miR-122-3p | 2.4 | hsa-miR-122-3p | 2.6 | | hsa-miR-122-3p | 2.6 | |
| hsa-miR-122-5p | 13.9 | hsa-miR-122-5p | 8.0 | hsa-miR-122-5p | 4.8 | | hsa-miR-122-5p | 4.0 | |
| hsa-miR-1228-3p | 4.3 | hsa-miR-1228-3p | 3.5 | hsa-miR-1228-3p | 4.0 | | hsa-miR-1228-3p | 5.1 | |
| hsa-miR-1229-5p | 3.2 | hsa-miR-1229-5p | 3.7 | hsa-miR-1234-3p | 4.2 | | hsa-miR-1229-5p | 4.9 | |
| hsa-miR-1234-3p | 4.3 | hsa-miR-1234-3p | 3.8 | hsa-miR-1237-3p | 3.1 | | hsa-miR-1234-3p | 5.1 | |
| hsa-miR-1237-3p | 3.3 | hsa-miR-1237-3p | 2.9 | hsa-miR-1238-3p | 3.6 | | hsa-miR-1237-3p | 4.0 | |
| hsa-miR-1238-3p | 4.0 | hsa-miR-1238-3p | 3.2 | hsa-miR-1246 | 6.1 | | hsa-miR-1238-3p | 4.7 | |
| hsa-miR-1246 | 7.7 | hsa-miR-1246 | 9.8 | hsa-miR-1249-3p | 3.1 | | hsa-miR-1246 | 7.7 | |
| hsa-miR-1249-3p | 3.8 | hsa-miR-1249-3p | 3.2 | hsa-miR-125a-5p | 6.6 | | hsa-miR-1249-3p | 3.8 | |
| hsa-miR-125a-5p | 3.6 | hsa-miR-125a-5p | 6.4 | hsa-miR-125b-5p | 7.9 | | hsa-miR-125a-5p | 7.3 | |
| hsa-miR-125b-5p | 8.0 | hsa-miR-125b-5p | 6.3 | hsa-miR-1260a | 7.9 | | hsa-miR-125b-5p | 9.0 | |
| hsa-miR-1260a | 7.7 | hsa-miR-1260a | 7.1 | hsa-miR-1260b | 7.9 | | hsa-miR-1260a | 9.7 | |
| hsa-miR-1260b | 6.8 | hsa-miR-1260b | 6.0 | hsa-miR-126-3p | 7.8 | | hsa-miR-1260b | 9.5 | |
| hsa-miR-126-3p | 8.2 | hsa-miR-126-3p | 2.6 | hsa-miR-1268a | 4.3 | | hsa-miR-126-3p | 11.2 | |
| hsa-miR-126-5p | 3.0 | hsa-miR-1268a | 6.0 | hsa-miR-1273g-3p | 9.8 | | hsa-miR-126-5p | 5.1 | |
| hsa-miR-1268a | 4.7 | hsa-miR-1273g-3p | 9.2 | hsa-miR-1275 | 4.1 | | hsa-miR-1268a | 5.4 | |
| hsa-miR-1273g-3p | 10.1 | hsa-miR-1275 | 3.5 | hsa-miR-1281 | 3.5 | | hsa-miR-1273g-3p | 10.7 | |
| hsa-miR-1275 | 3.6 | hsa-miR-1281 | 3.1 | hsa-miR-128-3p | 2.9 | | hsa-miR-1275 | 5.1 | |
| hsa-miR-1281 | 3.7 | hsa-miR-128-3p | 2.9 | hsa-miR-1290 | 4.1 | | hsa-miR-1281 | 4.5 | |
| hsa-miR-128-3p | 3.5 | hsa-miR-1290 | 7.2 | hsa-miR-129-2-3p | 3.1 | | hsa-miR-128-3p | 2.9 | |
| hsa-miR-1290 | 5.6 | hsa-miR-129-2-3p | 2.7 | hsa-miR-1304-3p | 3.9 | | hsa-miR-1290 | 5.4 | |
| hsa-miR-129-2-3p | 2.9 | hsa-miR-1304-3p | 3.3 | hsa-miR-1305 | 3.1 | | hsa-miR-129-2-3p | 3.3 | |
| hsa-miR-1304-3p | 4.0 | hsa-miR-1305 | 2.8 | hsa-miR-130a-3p | 8.7 | | hsa-miR-1304-3p | 4.9 | |
| hsa-miR-1305 | 3.7 | hsa-miR-130a-3p | 5.3 | hsa-miR-130b-3p | 5.2 | | hsa-miR-1305 | 3.7 | |
| hsa-miR-130a-3p | 7.2 | hsa-miR-130b-3p | 3.6 | hsa-miR-140-3p | 4.2 | | hsa-miR-130a-3p | 7.6 | |
| hsa-miR-130b-3p | 4.9 | hsa-miR-140-3p | 4.5 | hsa-miR-140-5p | 4.7 | | hsa-miR-130b-3p | 6.0 | |
| hsa-miR-140-3p | 4.7 | hsa-miR-140-5p | 6.0 | hsa-miR-142-3p | 5.7 | | hsa-miR-140-3p | 4.3 | |
| hsa-miR-140-5p | 5.1 | hsa-miR-142-3p | 9.4 | hsa-miR-145-5p | 7.4 | | hsa-miR-140-5p | 3.4 | |
| hsa-miR-145-5p | 4.3 | hsa-miR-145-5p | 3.2 | hsa-miR-146a-5p | 5.8 | | hsa-miR-145-5p | 6.8 | |
| hsa-miR-146a-5p | 3.8 | hsa-miR-146a-5p | 8.5 | hsa-miR-146b-5p | 4.1 | | hsa-miR-146a-5p | 4.3 | |
| hsa-miR-148a-3p | 8.4 | hsa-miR-146b-5p | 5.8 | hsa-miR-148a-3p | 4.7 | | hsa-miR-148a-3p | 3.4 | |
| hsa-miR-148b-3p | 3.7 | hsa-miR-148a-3p | 3.4 | hsa-miR-148b-3p | 3.2 | | hsa-miR-148b-3p | 3.1 | |
| hsa-miR-149-5p | 3.5 | hsa-miR-148b-3p | 3.8 | hsa-miR-149-5p | 4.3 | | hsa-miR-149-5p | 4.5 | |
| hsa-miR-150-5p | 2.6 | hsa-miR-149-5p | 3.2 | hsa-miR-150-5p | 4.0 | | hsa-miR-150-5p | 2.9 | |
| hsa-miR-151a-3p | 4.0 | hsa-miR-150-5p | 2.4 | hsa-miR-151a-3p | 4.6 | | hsa-miR-151a-3p | 5.5 | |
| hsa-miR-151a-5p | 6.5 | hsa-miR-151a-3p | 2.7 | hsa-miR-151a-5p | 6.5 | | hsa-miR-151a-5p | 6.5 | |
| hsa-miR-151b | 5.0 | hsa-miR-151a-5p | 4.4 | hsa-miR-151b | 4.8 | | hsa-miR-151b | 4.7 | |
| hsa-miR-152-3p | 3.2 | hsa-miR-151b | 3.6 | hsa-miR-152-3p | 2.6 | | hsa-miR-152-3p | 3.0 | |
| hsa-miR-1539 | 3.4 | hsa-miR-1539 | 3.0 | hsa-miR-1539 | 3.3 | | hsa-miR-1539 | 4.2 | |
| hsa-miR-155-5p | 3.7 | hsa-miR-155-5p | 6.2 | hsa-miR-155-5p | 5.1 | | hsa-miR-155-5p | 5.5 | |
| hsa-miR-15a-5p | 6.9 | hsa-miR-15a-5p | 8.4 | hsa-miR-15a-5p | 6.3 | | hsa-miR-15a-5p | 5.4 | |
| hsa-miR-15b-5p | 7.8 | hsa-miR-15b-5p | 8.6 | hsa-miR-15b-5p | 8.9 | | hsa-miR-15b-5p | 8.2 | |
| hsa-miR-16-5p | 8.5 | hsa-miR-16-5p | 9.4 | hsa-miR-16-5p | 8.6 | | hsa-miR-16-5p | 8.9 | |
| hsa-miR-17-5p | 6.3 | hsa-miR-17-5p | 5.5 | hsa-miR-17-3p | 2.9 | | hsa-miR-17-5p | 7.6 | |
| hsa-miR-181a-5p | 4.5 | hsa-miR-181a-5p | 6.4 | hsa-miR-17-5p | 6.8 | | hsa-miR-181a-5p | 5.7 | |
| hsa-miR-1825 | 3.7 | hsa-miR-1825 | 3.1 | hsa-miR-181a-5p | 5.8 | | hsa-miR-1825 | 4.3 | |
| hsa-miR-185-5p | 4.5 | hsa-miR-185-5p | 5.6 | hsa-miR-1825 | 3.1 | | hsa-miR-185-5p | 4.5 | |
| hsa-miR-186-5p | 3.7 | hsa-miR-186-5p | 3.0 | hsa-miR-185-5p | 4.0 | | hsa-miR-186-5p | 3.3 | |
| hsa-miR-188-5p | 3.0 | hsa-miR-188-5p | 3.6 | hsa-miR-186-5p | 2.7 | | hsa-miR-188-5p | 4.3 | |
| hsa-miR-18a-5p | 3.0 | hsa-miR-18a-5p | 2.8 | hsa-miR-18a-5p | 3.9 | | hsa-miR-18a-5p | 4.5 | |
| hsa-miR-1908-3p | 3.3 | hsa-miR-1908-3p | 2.8 | hsa-miR-1908-3p | 2.9 | | hsa-miR-1908-3p | 4.0 | |
| hsa-miR-191-3p | 3.4 | hsa-miR-191-3p | 2.9 | hsa-miR-191-3p | 3.2 | | hsa-miR-191-3p | 4.3 | |
| hsa-miR-1915-3p | 4.7 | hsa-miR-1914-3p | 3.4 | hsa-miR-1914-3p | 3.2 | | hsa-miR-1915-3p | 6.1 | |
| hsa-miR-192-5p | 9.8 | hsa-miR-1915-3p | 2.9 | hsa-miR-1915-3p | 4.3 | | hsa-miR-192-5p | 3.0 | |
| hsa-miR-193a-3p | 5.1 | hsa-miR-192-5p | 4.4 | hsa-miR-192-5p | 3.2 | | hsa-miR-193a-3p | 4.8 | |
| hsa-miR-193a-5p | 3.4 | hsa-miR-193a-3p | 3.7 | hsa-miR-193a-3p | 3.9 | | hsa-miR-193a-5p | 4.3 | |
| hsa-miR-193b-3p | 6.2 | hsa-miR-193a-5p | 4.4 | hsa-miR-193a-5p | 3.0 | | hsa-miR-193b-3p | 4.0 | |
| hsa-miR-194-5p | 8.0 | hsa-miR-194-5p | 2.3 | hsa-miR-194-5p | 2.4 | | hsa-miR-194-5p | 3.1 | |
| hsa-miR-195-5p | 6.3 | hsa-miR-193b-3p | 3.0 | hsa-miR-193b-3p | 4.0 | | hsa-miR-195-5p | 5.1 | |
| hsa-miR-1973 | 5.0 | hsa-miR-195-5p | 3.3 | hsa-miR-195-5p | 7.6 | | hsa-miR-1973 | 5.5 | |
| hsa-miR-197-3p | 3.9 | hsa-miR-1973 | 2.6 | hsa-miR-1973 | 3.3 | | hsa-miR-197-3p | 4.5 | |
| hsa-miR-197-5p | 6.3 | hsa-miR-197-3p | 5.0 | hsa-miR-197-3p | 4.3 | | hsa-miR-197-5p | 7.1 | |
| hsa-miR-199a-3p | 6.9 | hsa-miR-197-5p | 6.6 | hsa-miR-197-5p | 5.4 | | hsa-miR-199a-3p | 7.8 | |
| hsa-miR-199a-5p | 4.2 | hsa-miR-199a-3p | 7.0 | hsa-miR-199a-3p | 9.5 | | hsa-miR-199a-5p | 6.1 | |
| hsa-miR-19a-3p | 5.6 | hsa-miR-199a-5p | 4.0 | hsa-miR-199a-5p | 7.1 | | hsa-miR-19a-3p | 6.2 | |
| hsa-miR-19b-3p | 7.4 | hsa-miR-19a-3p | 6.4 | hsa-miR-19a-3p | 6.4 | | hsa-miR-19b-3p | 8.0 | |
| hsa-miR-200a-3p | 3.2 | hsa-miR-19b-3p | 7.5 | hsa-miR-19b-3p | 7.6 | | hsa-miR-200a-3p | 3.2 | |
| hsa-miR-200b-3p | 6.1 | hsa-miR-20a-5p | 7.1 | hsa-miR-200b-3p | 4.0 | | hsa-miR-200b-3p | 5.9 | |
| hsa-miR-20a-5p | 8.0 | hsa-miR-20b-5p | 4.2 | hsa-miR-20a-5p | 8.1 | | hsa-miR-20a-5p | 7.9 | |
| hsa-miR-20b-5p | 5.1 | hsa-miR-210-3p | 2.8 | hsa-miR-20b-5p | 4.9 | | hsa-miR-20b-5p | 5.1 | |
| hsa-miR-2116-3p | 3.4 | hsa-miR-2116-3p | 3.0 | hsa-miR-2116-3p | 3.2 | | hsa-miR-2116-3p | 4.1 | |
| hsa-miR-21-3p | 4.1 | hsa-miR-21-3p | 3.1 | hsa-miR-21-3p | 4.4 | | hsa-miR-21-3p | 4.2 | |
| hsa-miR-214-3p | 4.2 | hsa-miR-214-3p | 4.7 | hsa-miR-214-3p | 7.7 | | hsa-miR-214-3p | 5.6 | |
| hsa-miR-21-5p | 12.0 | hsa-miR-21-5p | 13.9 | hsa-miR-21-5p | 12.0 | | hsa-miR-21-5p | 11.4 | |
| hsa-miR-215-5p | 8.7 | hsa-miR-215-5p | 2.7 | hsa-miR-215-5p | 2.6 | | hsa-miR-215-5p | 3.0 | |
| hsa-miR-221-3p | 3.7 | hsa-miR-221-3p | 5.6 | hsa-miR-221-3p | 5.2 | | hsa-miR-221-3p | 6.9 | |
| hsa-miR-223-3p | 4.9 | hsa-miR-223-3p | 9.5 | hsa-miR-223-3p | 4.4 | | hsa-miR-223-3p | 3.7 | |
| hsa-miR-22-3p | 9.6 | hsa-miR-22-3p | 9.1 | hsa-miR-22-3p | 8.8 | | hsa-miR-22-3p | 8.7 | |
| hsa-miR-224-5p | 4.1 | hsa-miR-22-5p | 3.5 | hsa-miR-224-5p | 2.7 | | hsa-miR-224-5p | 3.0 | |
| hsa-miR-22-5p | 3.1 | hsa-miR-23a-3p | 10.2 | hsa-miR-22-5p | 2.9 | | hsa-miR-22-5p | 3.1 | |
| hsa-miR-23a-3p | 8.2 | hsa-miR-23b-3p | 5.3 | hsa-miR-23a-3p | 9.7 | | hsa-miR-23a-3p | 9.8 | |
| hsa-miR-23b-3p | 9.2 | hsa-miR-24-3p | 5.6 | hsa-miR-23b-3p | 7.2 | | hsa-miR-23b-3p | 6.7 | |
| hsa-miR-24-3p | 8.8 | hsa-miR-25-3p | 5.7 | hsa-miR-24-3p | 9.4 | | hsa-miR-24-3p | 9.8 | |
| hsa-miR-25-3p | 6.6 | hsa-miR-26a-5p | 7.0 | hsa-miR-25-3p | 6.8 | | hsa-miR-25-3p | 7.1 | |
| hsa-miR-26a-5p | 8.1 | hsa-miR-26b-5p | 8.5 | hsa-miR-26a-5p | 7.3 | | hsa-miR-26a-5p | 7.7 | |
| hsa-miR-26b-5p | 8.7 | hsa-miR-27a-3p | 9.3 | hsa-miR-26b-5p | 7.4 | | hsa-miR-26b-5p | 6.2 | |
| hsa-miR-27a-3p | 7.2 | hsa-miR-27b-3p | 5.8 | hsa-miR-27a-3p | 9.3 | | hsa-miR-27a-3p | 8.8 | |
| hsa-miR-27b-3p | 8.6 | hsa-miR-28-5p | 4.3 | hsa-miR-27b-3p | 7.5 | | hsa-miR-27b-3p | 6.7 | |
| hsa-miR-28-5p | 5.6 | hsa-miR-2861 | 3.2 | hsa-miR-28-5p | 5.6 | | hsa-miR-28-5p | 3.9 | |
| hsa-miR-2861 | 3.8 | hsa-miR-29a-3p | 10.1 | hsa-miR-2861 | 3.4 | | hsa-miR-2861 | 4.7 | |
| hsa-miR-29a-3p | 9.8 | hsa-miR-29b-1-5p | 2.9 | hsa-miR-29a-3p | 9.4 | | hsa-miR-29a-3p | 9.5 | |
| hsa-miR-29b-3p | 7.2 | hsa-miR-29b-3p | 8.5 | hsa-miR-29b-1-5p | 3.5 | | hsa-miR-29b-3p | 7.7 | |
| hsa-miR-29c-3p | 8.3 | hsa-miR-29c-3p | 8.6 | hsa-miR-29b-3p | 7.4 | | hsa-miR-29c-3p | 7.3 | |
| hsa-miR-30a-3p | 4.4 | hsa-miR-29c-5p | 2.8 | hsa-miR-29c-3p | 7.8 | | hsa-miR-30a-3p | 3.8 | |
| hsa-miR-30a-5p | 6.5 | hsa-miR-30a-5p | 4.0 | hsa-miR-30a-3p | 3.6 | | hsa-miR-30a-5p | 6.6 | |
| hsa-miR-30b-5p | 7.5 | hsa-miR-30b-5p | 5.7 | hsa-miR-30a-5p | 5.9 | | hsa-miR-30b-5p | 5.8 | |
| hsa-miR-30c-5p | 5.7 | hsa-miR-30c-5p | 4.7 | hsa-miR-30b-5p | 5.4 | | hsa-miR-30c-5p | 4.6 | |
| hsa-miR-30d-5p | 6.1 | hsa-miR-30d-5p | 5.4 | hsa-miR-30c-5p | 4.3 | | hsa-miR-30d-5p | 5.6 | |
| hsa-miR-30e-5p | 5.4 | hsa-miR-30e-3p | 3.2 | hsa-miR-30d-5p | 4.7 | | hsa-miR-30e-5p | 3.5 | |
| hsa-miR-3151-3p | 3.1 | hsa-miR-30e-5p | 5.4 | hsa-miR-30e-3p | 2.7 | | hsa-miR-3151-3p | 3.6 | |
| hsa-miR-3162-3p | 4.3 | hsa-miR-3151-3p | 2.7 | hsa-miR-30e-5p | 3.9 | | hsa-miR-3162-3p | 5.0 | |
| hsa-miR-3162-5p | 5.5 | hsa-miR-3162-3p | 3.5 | hsa-miR-3151-3p | 2.8 | | hsa-miR-3162-5p | 8.4 | |
| hsa-miR-3190-5p | 3.0 | hsa-miR-3162-5p | 7.6 | hsa-miR-3162-3p | 4.2 | | hsa-miR-3190-5p | 3.6 | |
| hsa-miR-3195 | 3.7 | hsa-miR-3190-5p | 2.7 | hsa-miR-3162-5p | 6.3 | | hsa-miR-3195 | 5.7 | |
| hsa-miR-3196 | 3.1 | hsa-miR-3195 | 2.6 | hsa-miR-3190-5p | 2.9 | | hsa-miR-3196 | 4.8 | |
| hsa-miR-3198 | 4.2 | hsa-miR-3196 | 2.9 | hsa-miR-3195 | 2.9 | | hsa-miR-3198 | 4.1 | |
| hsa-miR-320a | 5.6 | hsa-miR-3198 | 5.7 | hsa-miR-3198 | 4.7 | | hsa-miR-320a | 5.9 | |
| hsa-miR-320b | 6.6 | hsa-miR-320a | 6.2 | hsa-miR-320a | 5.2 | | hsa-miR-320b | 6.6 | |
| hsa-miR-320c | 5.9 | hsa-miR-320b | 5.1 | hsa-miR-320b | 5.9 | | hsa-miR-320c | 5.5 | |
| hsa-miR-320d | 7.1 | hsa-miR-320c | 4.5 | hsa-miR-320c | 4.8 | | hsa-miR-320d | 7.0 | |
| hsa-miR-320e | 6.6 | hsa-miR-320d | 6.0 | hsa-miR-320d | 6.2 | | hsa-miR-320e | 5.6 | |
| hsa-miR-324-3p | 5.7 | hsa-miR-320e | 4.8 | hsa-miR-320e | 5.4 | | hsa-miR-324-3p | 6.7 | |
| hsa-miR-324-5p | 3.8 | hsa-miR-324-3p | 6.0 | hsa-miR-324-3p | 5.9 | | hsa-miR-324-5p | 4.7 | |
| hsa-miR-331-3p | 6.2 | hsa-miR-324-5p | 4.3 | hsa-miR-324-5p | 4.5 | | hsa-miR-331-3p | 5.7 | |
| hsa-miR-33b-3p | 3.2 | hsa-miR-331-3p | 5.4 | hsa-miR-331-3p | 5.7 | | hsa-miR-33b-3p | 4.0 | |
| hsa-miR-342-3p | 6.0 | hsa-miR-33b-3p | 2.8 | hsa-miR-33b-3p | 3.0 | | hsa-miR-342-3p | 5.4 | |
| hsa-miR-34a-5p | 8.7 | hsa-miR-340-3p | 3.2 | hsa-miR-342-3p | 5.6 | | hsa-miR-34a-5p | 7.6 | |
| hsa-miR-34b-5p | 4.7 | hsa-miR-342-3p | 7.4 | hsa-miR-34a-5p | 8.2 | | hsa-miR-34b-5p | 3.5 | |
| hsa-miR-361-3p | 3.5 | hsa-miR-34a-5p | 9.1 | hsa-miR-34b-5p | 3.3 | | hsa-miR-361-3p | 3.2 | |
| hsa-miR-361-5p | 5.2 | hsa-miR-34b-5p | 4.5 | hsa-miR-361-3p | 2.6 | | hsa-miR-361-5p | 4.9 | |
| hsa-miR-3651 | 5.2 | hsa-miR-361-3p | 3.2 | hsa-miR-361-5p | 4.6 | | hsa-miR-3651 | 6.3 | |
| hsa-miR-3653-3p | 5.1 | hsa-miR-361-5p | 4.7 | hsa-miR-3651 | 5.2 | | hsa-miR-3653-3p | 4.9 | |
| hsa-miR-3656 | 3.6 | hsa-miR-3651 | 3.3 | hsa-miR-3653-3p | 4.0 | | hsa-miR-3656 | 4.7 | |
| hsa-miR-365a-3p | 7.1 | hsa-miR-3653-3p | 4.0 | hsa-miR-365a-3p | 5.3 | | hsa-miR-365a-3p | 5.9 | |
| hsa-miR-3663-3p | 5.7 | hsa-miR-3656 | 4.0 | hsa-miR-3663-3p | 6.0 | | hsa-miR-3663-3p | 6.0 | |
| hsa-miR-3665 | 4.5 | hsa-miR-365a-3p | 5.7 | hsa-miR-3665 | 3.6 | | hsa-miR-3665 | 4.8 | |
| hsa-miR-3679-5p | 3.3 | hsa-miR-3663-3p | 3.1 | hsa-miR-3679-5p | 3.2 | | hsa-miR-3679-5p | 4.6 | |
| hsa-miR-374a-5p | 5.3 | hsa-miR-3679-5p | 5.3 | hsa-miR-374a-5p | 5.0 | | hsa-miR-374a-5p | 5.0 | |
| hsa-miR-374b-5p | 5.0 | hsa-miR-374a-5p | 6.2 | hsa-miR-374b-5p | 4.2 | | hsa-miR-374b-5p | 5.2 | |
| hsa-miR-376a-3p | 3.0 | hsa-miR-374b-5p | 5.3 | hsa-miR-376a-3p | 5.7 | | hsa-miR-376a-3p | 5.7 | |
| hsa-miR-376c-3p | 3.4 | hsa-miR-376a-3p | 3.1 | hsa-miR-376c-3p | 6.1 | | hsa-miR-376c-3p | 6.0 | |
| hsa-miR-378a-3p | 5.8 | hsa-miR-376c-3p | 2.9 | hsa-miR-378a-3p | 4.1 | | hsa-miR-378a-3p | 4.0 | |
| hsa-miR-378i | 6.4 | hsa-miR-378a-3p | 6.0 | hsa-miR-378i | 4.6 | | hsa-miR-378i | 4.0 | |
| hsa-miR-3940-5p | 3.0 | hsa-miR-378a-5p | 2.8 | hsa-miR-3960 | 7.9 | | hsa-miR-3940-5p | 3.3 | |
| hsa-miR-3960 | 8.7 | hsa-miR-378d | 3.0 | hsa-miR-423-5p | 3.5 | | hsa-miR-3960 | 9.5 | |
| hsa-miR-423-5p | 3.8 | hsa-miR-378i | 6.6 | hsa-miR-424-5p | 7.3 | | hsa-miR-423-5p | 4.3 | |
| hsa-miR-424-5p | 4.4 | hsa-miR-3960 | 6.6 | hsa-miR-425-3p | 3.3 | | hsa-miR-424-5p | 5.2 | |
| hsa-miR-425-3p | 3.3 | hsa-miR-423-5p | 3.3 | hsa-miR-425-5p | 3.4 | | hsa-miR-425-3p | 4.3 | |
| hsa-miR-425-5p | 5.1 | hsa-miR-424-5p | 6.7 | hsa-miR-4281 | 8.9 | | hsa-miR-425-5p | 4.1 | |
| hsa-miR-4270 | 6.0 | hsa-miR-425-3p | 2.9 | hsa-miR-4284 | 8.5 | | hsa-miR-4270 | 4.2 | |
| hsa-miR-4271 | 3.2 | hsa-miR-425-5p | 4.2 | hsa-miR-4286 | 5.4 | | hsa-miR-4271 | 4.8 | |
| hsa-miR-4281 | 7.9 | hsa-miR-4270 | 3.4 | hsa-miR-4291 | 3.8 | | hsa-miR-4281 | 9.0 | |
| hsa-miR-4284 | 9.6 | hsa-miR-4271 | 5.4 | hsa-miR-4299 | 5.9 | | hsa-miR-4284 | 8.8 | |
| hsa-miR-4286 | 5.6 | hsa-miR-4281 | 6.5 | hsa-miR-4306 | 4.4 | | hsa-miR-4286 | 7.2 | |
| hsa-miR-4291 | 3.5 | hsa-miR-4284 | 6.9 | hsa-miR-4310 | 3.2 | | hsa-miR-4291 | 5.2 | |
| hsa-miR-4298 | 2.7 | hsa-miR-4286 | 6.2 | hsa-miR-4313 | 3.9 | | hsa-miR-4298 | 3.9 | |
| hsa-miR-4299 | 7.2 | hsa-miR-4291 | 3.4 | hsa-miR-4323 | 2.7 | | hsa-miR-4299 | 7.5 | |
| hsa-miR-4306 | 5.0 | hsa-miR-4298 | 5.5 | hsa-miR-4324 | 2.3 | | hsa-miR-4306 | 4.5 | |
| hsa-miR-4310 | 3.4 | hsa-miR-4299 | 5.0 | hsa-miR-4428 | 3.0 | | hsa-miR-4310 | 4.0 | |
| hsa-miR-4313 | 4.2 | hsa-miR-4306 | 6.3 | hsa-miR-4433a-5p | 3.0 | | hsa-miR-4313 | 5.1 | |
| hsa-miR-4323 | 2.8 | hsa-miR-4310 | 2.9 | hsa-miR-4442 | 3.6 | | hsa-miR-4323 | 3.1 | |
| hsa-miR-4324 | 2.4 | hsa-miR-4313 | 3.5 | hsa-miR-4443 | 3.0 | | hsa-miR-4324 | 2.5 | |
| hsa-miR-4428 | 3.0 | hsa-miR-4323 | 2.6 | hsa-miR-4459 | 8.1 | | hsa-miR-4428 | 5.2 | |
| hsa-miR-4433a-5p | 3.5 | hsa-miR-4428 | 3.0 | hsa-miR-4465 | 3.7 | | hsa-miR-4433a-5p | 4.1 | |
| hsa-miR-4442 | 3.7 | hsa-miR-4433a-5p | 2.9 | hsa-miR-4466 | 3.9 | | hsa-miR-4442 | 4.6 | |
| hsa-miR-4443 | 3.5 | hsa-miR-4442 | 4.1 | hsa-miR-4484 | 4.7 | | hsa-miR-4443 | 5.9 | |
| hsa-miR-4459 | 8.3 | hsa-miR-4443 | 3.2 | hsa-miR-4485-3p | 3.9 | | hsa-miR-4459 | 10.1 | |
| hsa-miR-4465 | 3.2 | hsa-miR-4459 | 7.9 | hsa-miR-4485-5p | 6.8 | | hsa-miR-4465 | 7.3 | |
| hsa-miR-4466 | 4.5 | hsa-miR-4465 | 4.8 | hsa-miR-4497 | 2.7 | | hsa-miR-4466 | 5.6 | |
| hsa-miR-4484 | 3.9 | hsa-miR-4466 | 3.0 | hsa-miR-4505 | 4.1 | | hsa-miR-4484 | 4.3 | |
| hsa-miR-4485-3p | 5.5 | hsa-miR-4484 | 6.0 | hsa-miR-4507 | 2.7 | | hsa-miR-4485-3p | 5.4 | |
| hsa-miR-4485-5p | 7.6 | hsa-miR-4485-3p | 3.2 | hsa-miR-4515 | 3.4 | | hsa-miR-4485-5p | 8.4 | |
| hsa-miR-4497 | 3.0 | hsa-miR-4485-5p | 6.9 | hsa-miR-4516 | 8.5 | | hsa-miR-4497 | 3.9 | |
| hsa-miR-4505 | 4.0 | hsa-miR-4505 | 4.5 | hsa-miR-4530 | 4.6 | | hsa-miR-4505 | 5.7 | |
| hsa-miR-4507 | 3.3 | hsa-miR-4507 | 2.9 | hsa-miR-455-3p | 7.4 | | hsa-miR-4507 | 4.2 | |
| hsa-miR-4515 | 3.7 | hsa-miR-4515 | 3.0 | hsa-miR-4634 | 2.9 | | hsa-miR-4515 | 4.6 | |
| hsa-miR-4516 | 9.9 | hsa-miR-4516 | 6.0 | hsa-miR-4649-3p | 3.6 | | hsa-miR-4516 | 9.7 | |
| hsa-miR-4530 | 5.2 | hsa-miR-4530 | 3.3 | hsa-miR-4652-3p | 2.9 | | hsa-miR-4530 | 7.2 | |
| hsa-miR-4532 | 2.8 | hsa-miR-455-3p | 3.5 | hsa-miR-4653-3p | 2.8 | | hsa-miR-4532 | 3.0 | |
| hsa-miR-455-3p | 6.6 | hsa-miR-4649-3p | 3.5 | hsa-miR-4665-3p | 4.3 | | hsa-miR-455-3p | 4.8 | |
| hsa-miR-4634 | 3.8 | hsa-miR-4652-3p | 2.7 | hsa-miR-4687-3p | 4.4 | | hsa-miR-4634 | 4.3 | |
| hsa-miR-4649-3p | 3.9 | hsa-miR-4665-3p | 3.6 | hsa-miR-4698 | 2.9 | | hsa-miR-4649-3p | 4.8 | |
| hsa-miR-4652-3p | 3.0 | hsa-miR-4687-3p | 3.9 | hsa-miR-4713-3p | 4.6 | | hsa-miR-4652-3p | 3.6 | |
| hsa-miR-4653-3p | 3.4 | hsa-miR-4698 | 2.8 | hsa-miR-4716-3p | 2.6 | | hsa-miR-4653-3p | 4.3 | |
| hsa-miR-4665-3p | 4.4 | hsa-miR-4713-3p | 5.5 | hsa-miR-4721 | 2.9 | | hsa-miR-4665-3p | 5.5 | |
| hsa-miR-4687-3p | 4.2 | hsa-miR-4721 | 3.6 | hsa-miR-4725-5p | 3.6 | | hsa-miR-4687-3p | 6.3 | |
| hsa-miR-4698 | 3.0 | hsa-miR-4725-5p | 3.1 | hsa-miR-4728-5p | 3.1 | | hsa-miR-4698 | 3.7 | |
| hsa-miR-4713-3p | 4.7 | hsa-miR-4728-5p | 3.9 | hsa-miR-4746-3p | 2.7 | | hsa-miR-4713-3p | 4.7 | |
| hsa-miR-4716-3p | 3.1 | hsa-miR-4749-3p | 3.0 | hsa-miR-4749-3p | 3.2 | | hsa-miR-4716-3p | 3.1 | |
| hsa-miR-4721 | 3.1 | hsa-miR-4758-3p | 2.8 | hsa-miR-4758-3p | 3.1 | | hsa-miR-4721 | 5.3 | |
| hsa-miR-4725-5p | 3.7 | hsa-miR-4763-3p | 2.8 | hsa-miR-4763-3p | 3.4 | | hsa-miR-4725-5p | 4.6 | |
| hsa-miR-4728-5p | 3.4 | hsa-miR-4769-3p | 2.7 | hsa-miR-4769-3p | 2.7 | | hsa-miR-4728-5p | 5.2 | |
| hsa-miR-4739 | 3.8 | hsa-miR-4787-3p | 3.0 | hsa-miR-4787-3p | 3.5 | | hsa-miR-4739 | 3.4 | |
| hsa-miR-4746-3p | 2.9 | hsa-miR-4787-5p | 3.0 | hsa-miR-4787-5p | 3.0 | | hsa-miR-4746-3p | 4.3 | |
| hsa-miR-4749-3p | 3.3 | hsa-miR-4788 | 3.2 | hsa-miR-484 | 2.9 | | hsa-miR-4749-3p | 4.1 | |
| hsa-miR-4758-3p | 3.3 | hsa-miR-4800-5p | 3.5 | hsa-miR-494-3p | 6.8 | | hsa-miR-4758-3p | 4.1 | |
| hsa-miR-4763-3p | 5.5 | hsa-miR-483-5p | 4.9 | hsa-miR-497-5p | 5.7 | | hsa-miR-4763-3p | 5.5 | |
| hsa-miR-4769-3p | 3.1 | hsa-miR-484 | 2.8 | hsa-miR-5006-5p | 4.4 | | hsa-miR-4769-3p | 3.8 | |
| hsa-miR-4778-5p | 3.1 | hsa-miR-494-3p | 5.3 | hsa-miR-505-3p | 3.6 | | hsa-miR-4778-5p | 3.6 | |
| hsa-miR-4787-3p | 3.4 | hsa-miR-5006-5p | 6.4 | hsa-miR-5100 | 8.0 | | hsa-miR-4787-3p | 4.4 | |
| hsa-miR-4787-5p | 3.4 | hsa-miR-505-3p | 3.4 | hsa-miR-513a-5p | 3.8 | | hsa-miR-4787-5p | 4.3 | |
| hsa-miR-4788 | 3.2 | hsa-miR-5100 | 7.9 | hsa-miR-532-5p | 3.4 | | hsa-miR-4788 | 3.8 | |
| hsa-miR-4800-5p | 4.8 | hsa-miR-513a-5p | 4.7 | hsa-miR-5581-5p | 2.9 | | hsa-miR-4800-5p | 3.9 | |
| hsa-miR-483-5p | 4.3 | hsa-miR-532-5p | 3.6 | hsa-miR-5739 | 6.1 | | hsa-miR-483-5p | 3.8 | |
| hsa-miR-484 | 3.4 | hsa-miR-5739 | 6.0 | hsa-miR-574-3p | 4.0 | | hsa-miR-484 | 3.7 | |
| hsa-miR-494-3p | 7.5 | hsa-miR-574-3p | 3.3 | hsa-miR-574-5p | 5.1 | | hsa-miR-494-3p | 10.1 | |
| hsa-miR-497-5p | 4.4 | hsa-miR-574-5p | 4.8 | hsa-miR-575 | 2.9 | | hsa-miR-497-5p | 3.4 | |
| hsa-miR-5006-5p | 3.3 | hsa-miR-5787 | 4.7 | hsa-miR-5787 | 4.3 | | hsa-miR-5006-5p | 5.0 | |
| hsa-miR-505-3p | 3.9 | hsa-miR-590-5p | 3.9 | hsa-miR-590-5p | 3.2 | | hsa-miR-505-3p | 3.8 | |
| hsa-miR-5100 | 7.8 | hsa-miR-6069 | 3.3 | hsa-miR-6069 | 3.9 | | hsa-miR-5100 | 9.7 | |
| hsa-miR-513a-5p | 3.0 | hsa-miR-6085 | 6.2 | hsa-miR-6085 | 6.3 | | hsa-miR-513a-5p | 5.2 | |
| hsa-miR-532-5p | 3.2 | hsa-miR-6087 | 6.1 | hsa-miR-6087 | 6.8 | | hsa-miR-532-5p | 3.5 | |
| hsa-miR-5581-5p | 3.1 | hsa-miR-6088 | 4.6 | hsa-miR-6088 | 5.3 | | hsa-miR-5581-5p | 3.3 | |
| hsa-miR-5703 | 3.5 | hsa-miR-6089 | 6.5 | hsa-miR-6089 | 7.6 | | hsa-miR-5703 | 3.5 | |
| hsa-miR-5739 | 5.9 | hsa-miR-6090 | 5.8 | hsa-miR-6090 | 7.6 | | hsa-miR-5739 | 7.2 | |
| hsa-miR-574-3p | 4.8 | hsa-miR-6124 | 5.2 | hsa-miR-6124 | 3.9 | | hsa-miR-574-3p | 4.8 | |
| hsa-miR-574-5p | 5.3 | hsa-miR-6125 | 6.5 | hsa-miR-6125 | 5.5 | | hsa-miR-574-5p | 6.7 | |
| hsa-miR-575 | 3.0 | hsa-miR-6127 | 8.7 | hsa-miR-6127 | 8.0 | | hsa-miR-575 | 4.7 | |
| hsa-miR-5787 | 4.9 | hsa-miR-6131 | 3.1 | hsa-miR-6131 | 3.5 | | hsa-miR-5787 | 6.7 | |
| hsa-miR-6068 | 2.9 | hsa-miR-6165 | 7.5 | hsa-miR-6165 | 6.1 | | hsa-miR-6068 | 3.3 | |
| hsa-miR-6069 | 4.0 | hsa-miR-638 | 2.9 | hsa-miR-638 | 3.1 | | hsa-miR-6069 | 4.9 | |
| hsa-miR-6085 | 6.0 | hsa-miR-642a-3p | 6.8 | hsa-miR-642a-3p | 5.8 | | hsa-miR-6085 | 7.6 | |
| hsa-miR-6087 | 6.8 | hsa-miR-642b-3p | 4.7 | hsa-miR-642b-3p | 3.1 | | hsa-miR-6087 | 8.7 | |
| hsa-miR-6088 | 6.0 | hsa-miR-6508-5p | 3.2 | hsa-miR-6508-5p | 3.5 | | hsa-miR-6088 | 6.7 | |
| hsa-miR-6089 | 7.6 | hsa-miR-6510-5p | 4.6 | hsa-miR-6515-3p | 3.5 | | hsa-miR-6089 | 9.1 | |
| hsa-miR-6090 | 7.8 | hsa-miR-6515-3p | 3.2 | hsa-miR-660-5p | 3.7 | | hsa-miR-6090 | 8.8 | |
| hsa-miR-6124 | 4.5 | hsa-miR-660-5p | 4.8 | hsa-miR-664b-3p | 3.4 | | hsa-miR-6124 | 5.7 | |
| hsa-miR-6125 | 4.9 | hsa-miR-664b-3p | 2.9 | hsa-miR-6717-5p | 3.4 | | hsa-miR-6125 | 6.3 | |
| hsa-miR-6127 | 7.6 | hsa-miR-6717-5p | 2.8 | hsa-miR-6724-5p | 3.1 | | hsa-miR-6127 | 8.1 | |
| hsa-miR-6131 | 4.1 | hsa-miR-6724-5p | 4.3 | hsa-miR-6731-3p | 3.1 | | hsa-miR-6131 | 4.2 | |
| hsa-miR-6165 | 5.0 | hsa-miR-6731-3p | 2.8 | hsa-miR-6734-5p | 2.9 | | hsa-miR-6165 | 6.0 | |
| hsa-miR-630 | 4.2 | hsa-miR-6734-5p | 3.2 | hsa-miR-6737-3p | 3.9 | | hsa-miR-630 | 4.1 | |
| hsa-miR-638 | 4.1 | hsa-miR-6737-3p | 3.4 | hsa-miR-6740-5p | 4.6 | | hsa-miR-638 | 5.0 | |
| hsa-miR-642a-3p | 5.9 | hsa-miR-6740-5p | 4.7 | hsa-miR-6749-5p | 5.7 | | hsa-miR-642a-3p | 8.2 | |
| hsa-miR-642b-3p | 4.4 | hsa-miR-6749-5p | 6.3 | hsa-miR-6752-3p | 3.2 | | hsa-miR-642b-3p | 4.7 | |
| hsa-miR-6508-5p | 3.7 | hsa-miR-6752-3p | 2.9 | hsa-miR-6763-3p | 3.1 | | hsa-miR-6508-5p | 4.7 | |
| hsa-miR-6515-3p | 3.8 | hsa-miR-6763-3p | 2.9 | hsa-miR-6763-5p | 3.8 | | hsa-miR-6515-3p | 4.5 | |
| hsa-miR-660-5p | 3.6 | hsa-miR-6763-5p | 5.5 | hsa-miR-6765-3p | 3.0 | | hsa-miR-660-5p | 3.5 | |
| hsa-miR-664b-3p | 3.6 | hsa-miR-6765-3p | 2.8 | hsa-miR-6767-5p | 2.7 | | hsa-miR-664b-3p | 3.9 | |
| hsa-miR-6717-5p | 3.9 | hsa-miR-6766-3p | 2.8 | hsa-miR-6777-3p | 3.1 | | hsa-miR-6717-5p | 4.0 | |
| hsa-miR-6724-5p | 3.2 | hsa-miR-6767-5p | 3.1 | hsa-miR-6780b-5p | 4.1 | | hsa-miR-6724-5p | 5.1 | |
| hsa-miR-6731-3p | 3.1 | hsa-miR-6769b-5p | 3.6 | hsa-miR-6785-3p | 3.0 | | hsa-miR-6731-3p | 3.9 | |
| hsa-miR-6734-5p | 3.4 | hsa-miR-6777-3p | 2.8 | hsa-miR-6785-5p | 3.4 | | hsa-miR-6734-5p | 4.0 | |
| hsa-miR-6737-3p | 4.1 | hsa-miR-6780b-5p | 4.6 | hsa-miR-6797-3p | 3.6 | | hsa-miR-6737-3p | 4.9 | |
| hsa-miR-6740-5p | 5.0 | hsa-miR-6785-3p | 2.9 | hsa-miR-6800-3p | 3.4 | | hsa-miR-6740-5p | 5.5 | |
| hsa-miR-6749-5p | 5.9 | hsa-miR-6785-5p | 2.9 | hsa-miR-6800-5p | 5.5 | | hsa-miR-6749-5p | 7.6 | |
| hsa-miR-6752-3p | 3.2 | hsa-miR-6797-3p | 3.2 | hsa-miR-6803-5p | 3.2 | | hsa-miR-6752-3p | 4.0 | |
| hsa-miR-6763-3p | 3.3 | hsa-miR-6800-3p | 3.2 | hsa-miR-6812-3p | 3.0 | | hsa-miR-6763-3p | 4.1 | |
| hsa-miR-6763-5p | 3.1 | hsa-miR-6800-5p | 4.2 | hsa-miR-6813-3p | 3.5 | | hsa-miR-6763-5p | 4.1 | |
| hsa-miR-6765-3p | 3.2 | hsa-miR-6803-5p | 3.0 | hsa-miR-6819-3p | 4.2 | | hsa-miR-6765-3p | 3.7 | |
| hsa-miR-6766-3p | 3.2 | hsa-miR-6812-3p | 2.8 | hsa-miR-6821-5p | 5.9 | | hsa-miR-6766-3p | 3.8 | |
| hsa-miR-6767-5p | 3.1 | hsa-miR-6813-3p | 3.1 | hsa-miR-6824-3p | 3.3 | | hsa-miR-6767-5p | 3.3 | |
| hsa-miR-6769b-5p | 3.3 | hsa-miR-6819-3p | 3.6 | hsa-miR-6826-5p | 3.4 | | hsa-miR-6769b-5p | 5.7 | |
| hsa-miR-6777-3p | 3.2 | hsa-miR-6821-5p | 4.3 | hsa-miR-6850-5p | 3.9 | | hsa-miR-6777-3p | 4.0 | |
| hsa-miR-6780b-5p | 4.5 | hsa-miR-6824-3p | 3.4 | hsa-miR-6858-3p | 3.2 | | hsa-miR-6780b-5p | 5.3 | |
| hsa-miR-6785-3p | 3.1 | hsa-miR-6826-5p | 3.5 | hsa-miR-6865-3p | 3.3 | | hsa-miR-6785-3p | 3.8 | |
| hsa-miR-6785-5p | 3.9 | hsa-miR-6858-3p | 2.8 | hsa-miR-6869-5p | 6.0 | | hsa-miR-6785-5p | 7.2 | |
| hsa-miR-6797-3p | 3.9 | hsa-miR-6865-3p | 2.9 | hsa-miR-6870-3p | 2.9 | | hsa-miR-6797-3p | 4.7 | |
| hsa-miR-6800-3p | 3.9 | hsa-miR-6867-5p | 3.7 | hsa-miR-6875-5p | 6.5 | | hsa-miR-6800-3p | 4.7 | |
| hsa-miR-6800-5p | 5.1 | hsa-miR-6869-5p | 4.1 | hsa-miR-6879-5p | 6.2 | | hsa-miR-6800-5p | 6.4 | |
| hsa-miR-6803-5p | 3.8 | hsa-miR-6870-3p | 2.6 | hsa-miR-6880-3p | 3.1 | | hsa-miR-6803-5p | 5.3 | |
| hsa-miR-6812-3p | 3.2 | hsa-miR-6875-5p | 5.8 | hsa-miR-6889-3p | 3.2 | | hsa-miR-6812-3p | 3.9 | |
| hsa-miR-6813-3p | 3.5 | hsa-miR-6879-5p | 7.8 | hsa-miR-6891-5p | 2.8 | | hsa-miR-6813-3p | 4.3 | |
| hsa-miR-6819-3p | 4.3 | hsa-miR-6880-3p | 2.8 | hsa-miR-6893-5p | 3.8 | | hsa-miR-6819-3p | 4.9 | |
| hsa-miR-6821-5p | 5.9 | hsa-miR-6889-3p | 3.1 | hsa-miR-7107-5p | 4.5 | | hsa-miR-6821-5p | 7.2 | |
| hsa-miR-6824-3p | 3.2 | hsa-miR-6891-5p | 3.7 | hsa-miR-7110-5p | 3.1 | | hsa-miR-6824-3p | 3.9 | |
| hsa-miR-6826-5p | 4.2 | hsa-miR-6893-5p | 5.4 | hsa-miR-7150 | 3.8 | | hsa-miR-6826-5p | 5.2 | |
| hsa-miR-6850-5p | 4.4 | hsa-miR-7107-5p | 6.2 | hsa-miR-762 | 5.3 | | hsa-miR-6850-5p | 4.8 | |
| hsa-miR-6858-3p | 3.2 | hsa-miR-7110-5p | 4.2 | hsa-miR-7641 | 8.0 | | hsa-miR-6858-3p | 4.0 | |
| hsa-miR-6865-3p | 3.4 | hsa-miR-7150 | 4.0 | hsa-miR-766-3p | 3.4 | | hsa-miR-6865-3p | 4.4 | |
| hsa-miR-6867-5p | 3.1 | hsa-miR-762 | 3.4 | hsa-miR-7704 | 3.3 | | hsa-miR-6867-5p | 3.8 | |
| hsa-miR-6869-5p | 5.6 | hsa-miR-7641 | 7.3 | hsa-miR-7975 | 13.4 | | hsa-miR-6869-5p | 7.9 | |
| hsa-miR-6870-3p | 2.8 | hsa-miR-765 | 3.3 | hsa-miR-7977 | 12.2 | | hsa-miR-6870-3p | 3.5 | |
| hsa-miR-6875-5p | 6.8 | hsa-miR-766-3p | 3.4 | hsa-miR-8069 | 10.2 | | hsa-miR-6875-5p | 7.3 | |
| hsa-miR-6879-5p | 5.6 | hsa-miR-7704 | 2.9 | hsa-miR-92a-3p | 6.9 | | hsa-miR-6879-5p | 6.6 | |
| hsa-miR-6880-3p | 3.2 | hsa-miR-7847-3p | 5.3 | hsa-miR-933 | 2.8 | | hsa-miR-6880-3p | 3.8 | |
| hsa-miR-6889-3p | 3.5 | hsa-miR-7975 | 12.7 | hsa-miR-93-5p | 5.6 | | hsa-miR-6889-3p | 4.6 | |
| hsa-miR-6891-5p | 3.1 | hsa-miR-7977 | 11.8 | hsa-miR-937-5p | 4.0 | | hsa-miR-6891-5p | 4.6 | |
| hsa-miR-6893-5p | 3.4 | hsa-miR-8069 | 10.0 | hsa-miR-940 | 4.7 | | hsa-miR-6893-5p | 5.6 | |
| hsa-miR-7107-5p | 4.5 | hsa-miR-8072 | 3.9 | hsa-miR-98-5p | 4.1 | | hsa-miR-7107-5p | 6.1 | |
| hsa-miR-7110-5p | 3.8 | hsa-miR-92a-3p | 5.6 | hsa-miR-99a-5p | 6.1 | | hsa-miR-7110-5p | 5.6 | |
| hsa-miR-7150 | 3.6 | hsa-miR-933 | 2.5 | hsa-miR-99b-5p | 6.1 | | hsa-miR-7150 | 5.5 | |
| hsa-miR-762 | 4.2 | hsa-miR-93-5p | 4.3 | hsa-miR-10b-5p | 4.1 | | hsa-miR-762 | 7.6 | |
| hsa-miR-7641 | 7.9 | hsa-miR-937-5p | 4.3 | hsa-miR-125a-3p | 2.6 | | hsa-miR-7641 | 11.0 | |
| hsa-miR-765 | 3.2 | hsa-miR-940 | 4.1 | hsa-miR-132-3p | 4.7 | | hsa-miR-765 | 3.3 | |
| hsa-miR-766-3p | 3.6 | hsa-miR-98-5p | 4.2 | hsa-miR-143-3p | 4.5 | | hsa-miR-766-3p | 4.3 | |
| hsa-miR-7704 | 3.7 | hsa-miR-99a-5p | 3.4 | hsa-miR-150-3p | 3.5 | | hsa-miR-7704 | 4.4 | |
| hsa-miR-7847-3p | 3.2 | hsa-miR-99b-5p | 5.4 | hsa-miR-1587 | 2.7 | | hsa-miR-7847-3p | 3.8 | |
| hsa-miR-7975 | 13.1 | hsa-miR-1268b | 4.9 | hsa-miR-222-3p | 3.4 | | hsa-miR-7975 | 14.7 | |
| hsa-miR-7977 | 11.6 | hsa-miR-132-3p | 5.2 | hsa-miR-3152-3p | 5.6 | | hsa-miR-7977 | 13.7 | |
| hsa-miR-8063 | 3.3 | hsa-miR-150-3p | 3.6 | hsa-miR-3180-5p | 2.9 | | hsa-miR-8063 | 4.3 | |
| hsa-miR-8069 | 11.4 | hsa-miR-1587 | 2.9 | hsa-miR-362-5p | 3.1 | | hsa-miR-8069 | 12.4 | |
| hsa-miR-8072 | 2.9 | hsa-miR-193b-5p | 3.2 | hsa-miR-4436b-5p | 3.0 | | hsa-miR-8072 | 4.5 | |
| hsa-miR-874-3p | 2.9 | hsa-miR-222-3p | 2.7 | hsa-miR-4664-3p | 2.9 | | hsa-miR-874-3p | 4.4 | |
| hsa-miR-885-5p | 6.8 | hsa-miR-885-5p | 2.4 | hsa-miR-885-5p | 2.4 | | hsa-miR-885-5p | 2.6 | |
| hsa-miR-92a-3p | 6.9 | hsa-miR-296-5p | 2.7 | hsa-miR-4701-5p | 2.9 | | hsa-miR-92a-3p | 7.5 | |
| hsa-miR-933 | 2.7 | hsa-miR-3141 | 5.0 | hsa-miR-532-3p | 3.1 | | hsa-miR-933 | 3.3 | |
| hsa-miR-93-5p | 5.3 | hsa-miR-3152-3p | 7.9 | hsa-miR-550a-5p | 3.1 | | hsa-miR-93-5p | 6.7 | |
| hsa-miR-937-5p | 4.2 | hsa-miR-3180-5p | 2.8 | hsa-miR-602 | 2.8 | | hsa-miR-937-5p | 4.8 | |
| hsa-miR-940 | 4.9 | hsa-miR-338-3p | 4.0 | hsa-miR-6126 | 2.8 | | hsa-miR-940 | 6.0 | |
| hsa-miR-98-5p | 3.9 | hsa-miR-362-5p | 3.4 | hsa-miR-671-5p | 4.9 | | hsa-miR-98-5p | 4.1 | |
| hsa-miR-99a-5p | 7.5 | hsa-miR-3646 | 2.7 | hsa-miR-6727-5p | 3.0 | | hsa-miR-99a-5p | 6.9 | |
| hsa-miR-99b-5p | 3.1 | hsa-miR-371a-5p | 2.8 | hsa-miR-6760-3p | 3.1 | | hsa-miR-99b-5p | 7.1 | |
| hsa-miR-10b-5p | 2.5 | hsa-miR-4436b-5p | 2.8 | hsa-miR-6848-3p | 2.9 | | hsa-miR-10b-5p | 4.1 | |
| hsa-miR-1227-3p | 2.5 | hsa-miR-451a | 3.0 | hsa-miR-6851-3p | 2.9 | | hsa-miR-1227-3p | 2.8 | |
| hsa-miR-125a-3p | 2.5 | hsa-miR-4664-3p | 2.8 | hsa-miR-7111-3p | 3.0 | | hsa-miR-125a-3p | 3.3 | |
| hsa-miR-1268b | 2.8 | hsa-miR-4669 | 4.2 | hsa-miR-7974 | 3.1 | | hsa-miR-1268b | 4.3 | |
| hsa-miR-132-3p | 2.6 | hsa-miR-4701-5p | 2.8 | hsa-miR-8485 | 3.0 | | hsa-miR-132-3p | 3.6 | |
| hsa-miR-135a-3p | 2.6 | hsa-miR-5001-5p | 4.0 | hsa-miR-1185-1-3p | 3.1 | | hsa-miR-135a-3p | 4.0 | |
| hsa-miR-143-3p | 2.5 | hsa-miR-505-5p | 2.5 | hsa-miR-1229-3p | 2.9 | | hsa-miR-143-3p | 4.1 | |
| hsa-miR-150-3p | 2.7 | hsa-miR-532-3p | 3.4 | hsa-miR-127-3p | 5.1 | | hsa-miR-150-3p | 4.7 | |
| hsa-miR-1587 | 2.6 | hsa-miR-550a-5p | 3.0 | hsa-miR-181b-5p | 4.1 | | hsa-miR-1587 | 4.4 | |
| hsa-miR-222-3p | 2.7 | hsa-miR-602 | 2.7 | hsa-miR-18b-5p | 2.4 | | hsa-miR-222-3p | 5.6 | |
| hsa-miR-296-5p | 2.9 | hsa-miR-671-5p | 6.9 | hsa-miR-23c | 3.0 | | hsa-miR-296-5p | 3.3 | |
| hsa-miR-3141 | 2.9 | hsa-miR-6752-5p | 3.4 | hsa-miR-3675-3p | 2.9 | | hsa-miR-3141 | 4.2 | |
| hsa-miR-3180-5p | 3.0 | hsa-miR-6756-5p | 5.0 | hsa-miR-487b-3p | 4.4 | | hsa-miR-3180-5p | 3.4 | |
| hsa-miR-362-5p | 2.9 | hsa-miR-6760-3p | 2.8 | hsa-miR-5010-3p | 2.8 | | hsa-miR-362-5p | 2.9 | |
| hsa-miR-3646 | 2.8 | hsa-miR-6798-3p | 2.7 | hsa-miR-652-3p | 2.6 | | hsa-miR-3646 | 3.1 | |
| hsa-miR-371a-5p | 2.2 | hsa-miR-6848-3p | 2.8 | hsa-miR-654-3p | 4.6 | | hsa-miR-371a-5p | 2.9 | |
| hsa-miR-4436b-5p | 3.2 | hsa-miR-6851-3p | 2.8 | hsa-miR-6760-5p | 5.0 | | hsa-miR-4436b-5p | 4.0 | |
| hsa-miR-4664-3p | 3.2 | hsa-miR-6858-5p | 3.0 | hsa-miR-6795-3p | 2.9 | | hsa-miR-4664-3p | 4.3 | |
| hsa-miR-4669 | 2.7 | hsa-miR-6861-3p | 2.7 | hsa-miR-7-5p | 3.6 | | hsa-miR-4669 | 4.3 | |
| hsa-miR-4701-5p | 3.1 | hsa-miR-6892-3p | 2.7 | hsa-miR-1181 | 2.6 | | hsa-miR-4701-5p | 3.5 | |
| hsa-miR-4741 | 2.5 | hsa-miR-7111-3p | 2.7 | hsa-miR-136-5p | 3.5 | | hsa-miR-4741 | 5.1 | |
| hsa-miR-5001-5p | 2.8 | hsa-miR-7974 | 2.8 | hsa-miR-145-3p | 3.5 | | hsa-miR-5001-5p | 3.1 | |
| hsa-miR-532-3p | 2.9 | hsa-miR-8485 | 3.7 | hsa-miR-154-3p | 3.9 | | hsa-miR-532-3p | 3.5 | |
| hsa-miR-550a-5p | 3.2 | hsa-miR-92b-3p | 2.5 | hsa-miR-154-5p | 2.9 | | hsa-miR-550a-5p | 4.0 | |
| hsa-miR-602 | 3.2 | hsa-miR-1185-1-3p | 3.4 | hsa-miR-181a-2-3p | 2.5 | | hsa-miR-602 | 3.9 | |
| hsa-miR-6126 | 2.7 | hsa-miR-1224-5p | 3.9 | hsa-miR-199b-5p | 2.4 | | hsa-miR-6126 | 5.1 | |
| hsa-miR-6132 | 3.1 | hsa-miR-1229-3p | 2.6 | hsa-miR-214-5p | 2.6 | | hsa-miR-6132 | 5.3 | |
| hsa-miR-671-5p | 2.8 | hsa-miR-1287-5p | 5.8 | hsa-miR-299-5p | 4.7 | | hsa-miR-671-5p | 5.8 | |
| hsa-miR-6727-5p | 3.4 | hsa-miR-129-1-3p | 2.6 | hsa-miR-31-5p | 2.5 | | hsa-miR-6727-5p | 4.9 | |
| hsa-miR-6752-5p | 2.7 | hsa-miR-181b-5p | 4.2 | hsa-miR-337-3p | 2.8 | | hsa-miR-6752-5p | 3.9 | |
| hsa-miR-6756-5p | 2.9 | hsa-miR-18b-5p | 2.3 | hsa-miR-337-5p | 3.3 | | hsa-miR-6756-5p | 4.2 | |
| hsa-miR-6760-3p | 3.1 | hsa-miR-2276-3p | 3.5 | hsa-miR-3613-3p | 2.7 | | hsa-miR-6760-3p | 3.9 | |
| hsa-miR-6798-3p | 2.8 | hsa-miR-2392 | 4.0 | hsa-miR-369-5p | 2.6 | | hsa-miR-6798-3p | 3.2 | |
| hsa-miR-6848-3p | 3.2 | hsa-miR-23c | 2.7 | hsa-miR-370-3p | 2.7 | | hsa-miR-6848-3p | 3.9 | |
| hsa-miR-6851-3p | 3.0 | hsa-miR-3124-5p | 4.1 | hsa-miR-376b-3p | 2.6 | | hsa-miR-6851-3p | 4.0 | |
| hsa-miR-6858-5p | 2.4 | hsa-miR-3127-5p | 4.4 | hsa-miR-377-3p | 3.9 | | hsa-miR-6858-5p | 3.2 | |
| hsa-miR-6861-3p | 3.0 | hsa-miR-3135b | 2.5 | hsa-miR-379-5p | 4.2 | | hsa-miR-6861-3p | 3.6 | |
| hsa-miR-6892-3p | 2.8 | hsa-miR-3156-5p | 4.0 | hsa-miR-381-3p | 4.9 | | hsa-miR-6892-3p | 3.2 | |
| hsa-miR-7108-5p | 2.8 | hsa-miR-340-5p | 4.3 | hsa-miR-382-5p | 3.0 | | hsa-miR-7108-5p | 4.1 | |
| hsa-miR-7111-3p | 2.7 | hsa-miR-342-5p | 3.0 | hsa-miR-409-3p | 5.2 | | hsa-miR-7111-3p | 3.6 | |
| hsa-miR-7974 | 3.3 | hsa-miR-362-3p | 3.6 | hsa-miR-409-5p | 2.6 | | hsa-miR-7974 | 3.7 | |
| hsa-miR-8485 | 2.7 | hsa-miR-3675-3p | 2.7 | hsa-miR-410-3p | 3.7 | | hsa-miR-8485 | 4.5 | |
| hsa-miR-92b-3p | 2.9 | hsa-miR-3682-3p | 6.7 | hsa-miR-411-5p | 2.6 | | hsa-miR-92b-3p | 3.4 | |
| hsa-miR-1185-1-3p | 2.5 | hsa-miR-3917 | 4.3 | hsa-miR-4312 | 2.8 | | hsa-miR-1185-1-3p | 4.1 | |
| hsa-miR-1224-5p | 2.4 | hsa-miR-3937 | 4.8 | hsa-miR-431-5p | 3.2 | | hsa-miR-1224-5p | 3.1 | |
| hsa-miR-1229-3p | 2.9 | hsa-miR-4257 | 5.5 | hsa-miR-432-5p | 4.1 | | hsa-miR-1229-3p | 3.3 | |
| hsa-miR-127-3p | 2.7 | hsa-miR-4327 | 2.9 | hsa-miR-485-3p | 2.9 | | hsa-miR-127-3p | 4.8 | |
| hsa-miR-129-1-3p | 2.8 | hsa-miR-4478 | 2.8 | hsa-miR-493-5p | 3.9 | | hsa-miR-129-1-3p | 3.1 | |
| hsa-miR-134-5p | 2.6 | hsa-miR-4486 | 3.6 | hsa-miR-495-3p | 3.9 | | hsa-miR-134-5p | 3.4 | |
| hsa-miR-181b-5p | 2.7 | hsa-miR-4496 | 3.8 | hsa-miR-503-5p | 2.9 | | hsa-miR-181b-5p | 5.1 | |
| hsa-miR-18b-5p | 2.3 | hsa-miR-4499 | 4.2 | hsa-miR-543 | 3.2 | | hsa-miR-18b-5p | 2.8 | |
| hsa-miR-2392 | 2.2 | hsa-miR-454-3p | 2.5 | hsa-miR-563 | 2.7 | | hsa-miR-2392 | 4.0 | |
| hsa-miR-23c | 2.9 | hsa-miR-4655-5p | 5.2 | hsa-miR-758-3p | 2.9 | | hsa-miR-23c | 3.6 | |
| hsa-miR-3135b | 2.4 | hsa-miR-4728-3p | 2.7 |  |  | | hsa-miR-3135b | 3.9 | |
| hsa-miR-3156-5p | 2.6 | hsa-miR-4732-5p | 3.0 |  |  | | hsa-miR-3156-5p | 3.3 | |
| hsa-miR-328-5p | 2.5 | hsa-miR-4793-5p | 3.1 |  |  | | hsa-miR-328-5p | 3.3 | |
| hsa-miR-3675-3p | 2.7 | hsa-miR-500a-3p | 3.3 |  |  | | hsa-miR-3675-3p | 3.5 | |
| hsa-miR-3679-3p | 2.9 | hsa-miR-5010-3p | 2.6 |  |  | | hsa-miR-3679-3p | 3.2 | |
| hsa-miR-4257 | 2.5 | hsa-miR-5196-5p | 6.9 |  |  | | hsa-miR-4257 | 3.6 | |
| hsa-miR-4327 | 2.8 | hsa-miR-584-5p | 5.2 |  |  | | hsa-miR-4327 | 3.8 | |
| hsa-miR-4455 | 2.4 | hsa-miR-6076 | 6.8 |  |  | | hsa-miR-4455 | 3.2 | |
| hsa-miR-4478 | 2.5 | hsa-miR-631 | 4.4 |  |  | | hsa-miR-4478 | 4.2 | |
| hsa-miR-4646-3p | 2.7 | hsa-miR-652-3p | 3.0 |  |  | | hsa-miR-4646-3p | 3.5 | |
| hsa-miR-4672 | 2.5 | hsa-miR-6739-5p | 3.1 |  |  | | hsa-miR-4672 | 3.4 | |
| hsa-miR-4728-3p | 2.9 | hsa-miR-6741-5p | 4.5 |  |  | | hsa-miR-4728-3p | 3.3 | |
| hsa-miR-4731-3p | 2.7 | hsa-miR-6757-3p | 2.8 |  |  | | hsa-miR-4731-3p | 3.1 | |
| hsa-miR-487b-3p | 2.7 | hsa-miR-6757-5p | 5.5 |  |  | | hsa-miR-487b-3p | 4.6 | |
| hsa-miR-5010-3p | 3.0 | hsa-miR-6760-5p | 7.0 |  |  | | hsa-miR-5010-3p | 3.7 | |
| hsa-miR-513b-5p | 2.5 | hsa-miR-6775-5p | 3.2 |  |  | | hsa-miR-513b-5p | 3.4 | |
| hsa-miR-5196-5p | 2.9 | hsa-miR-6791-5p | 4.3 |  |  | | hsa-miR-5196-5p | 5.6 | |
| hsa-miR-584-5p | 2.4 | hsa-miR-6794-5p | 3.5 |  |  | | hsa-miR-584-5p | 4.7 | |
| hsa-miR-6076 | 2.7 | hsa-miR-6795-3p | 2.6 |  |  | | hsa-miR-6076 | 5.5 | |
| hsa-miR-652-3p | 2.4 | hsa-miR-6808-5p | 4.5 |  |  | | hsa-miR-652-3p | 3.0 | |
| hsa-miR-654-3p | 2.6 | hsa-miR-6812-5p | 5.7 |  |  | | hsa-miR-654-3p | 4.2 | |
| hsa-miR-6728-5p | 2.5 | hsa-miR-6829-5p | 4.0 |  |  | | hsa-miR-6728-5p | 3.7 | |
| hsa-miR-6757-3p | 3.1 | hsa-miR-6892-5p | 4.6 |  |  | | hsa-miR-6757-3p | 3.6 | |
| hsa-miR-6757-5p | 2.4 | hsa-miR-7-5p | 3.4 |  |  | | hsa-miR-6757-5p | 3.5 | |
| hsa-miR-6775-5p | 2.4 | hsa-miR-8060 | 4.1 |  |  | | hsa-miR-6775-5p | 3.6 | |
| hsa-miR-6786-5p | 2.5 | hsa-miR-939-5p | 3.1 |  |  | | hsa-miR-6786-5p | 3.3 | |
| hsa-miR-6789-5p | 2.6 | hsa-miR-1236-5p | 3.5 |  |  | | hsa-miR-6789-5p | 3.5 | |
| hsa-miR-6791-5p | 2.6 | hsa-miR-1271-5p | 2.5 |  |  | | hsa-miR-6791-5p | 4.2 | |
| hsa-miR-6794-5p | 2.5 | hsa-miR-1273e | 3.4 |  |  | | hsa-miR-6794-5p | 3.4 | |
| hsa-miR-6795-3p | 3.0 | hsa-miR-1299 | 4.2 |  |  | | hsa-miR-6795-3p | 3.6 | |
| hsa-miR-6796-3p | 2.8 | hsa-miR-132-5p | 2.5 |  |  | | hsa-miR-6796-3p | 3.3 | |
| hsa-miR-6812-5p | 2.7 | hsa-miR-133b | 2.5 |  |  | | hsa-miR-6812-5p | 5.0 | |
| hsa-miR-6829-5p | 2.5 | hsa-miR-1-3p | 2.9 |  |  | | hsa-miR-6829-5p | 4.1 | |
| hsa-miR-6833-5p | 2.3 | hsa-miR-142-5p | 4.8 |  |  | | hsa-miR-6833-5p | 3.2 | |
| hsa-miR-6855-3p | 2.9 | hsa-miR-212-3p | 2.9 |  |  | | hsa-miR-6855-3p | 3.6 | |
| hsa-miR-718 | 2.6 | hsa-miR-221-5p | 2.6 |  |  | | hsa-miR-718 | 3.0 | |
| hsa-miR-7-5p | 2.4 | hsa-miR-3138 | 3.7 |  |  | | hsa-miR-7-5p | 2.9 | |
| hsa-miR-8060 | 2.3 | hsa-miR-31-3p | 2.9 |  |  | | hsa-miR-8060 | 4.2 | |
| hsa-miR-939-5p | 2.4 | hsa-miR-31-5p | 2.9 |  |  | | hsa-miR-939-5p | 3.8 | |
| hsa-miR-98-3p | 3.0 | hsa-miR-326 | 2.4 |  |  | | hsa-miR-98-3p | 3.4 | |
| hsa-miR-1181 | 2.6 | hsa-miR-3652 | 3.7 |  |  | | hsa-miR-1181 | 4.6 | |
| hsa-miR-1273e | 2.4 | hsa-miR-370-3p | 3.2 |  |  | | hsa-miR-1273e | 3.3 | |
| hsa-miR-139-5p | 2.3 | hsa-miR-3911 | 3.4 |  |  | | hsa-miR-139-5p | 3.9 | |
| hsa-miR-299-5p | 2.6 | hsa-miR-4253 | 3.4 |  |  | | hsa-miR-299-5p | 3.9 | |
| hsa-miR-337-5p | 2.3 | hsa-miR-4317 | 2.5 |  |  | | hsa-miR-337-5p | 3.3 | |
| hsa-miR-3613-3p | 3.0 | hsa-miR-4419a | 3.2 |  |  | | hsa-miR-3613-3p | 3.5 | |
| hsa-miR-3614-5p | 2.8 | hsa-miR-4430 | 2.9 |  |  | | hsa-miR-3614-5p | 3.3 | |
| hsa-miR-3620-3p | 2.9 | hsa-miR-4632-5p | 4.6 |  |  | | hsa-miR-3620-3p | 3.2 | |
| hsa-miR-3652 | 2.2 | hsa-miR-4687-5p | 2.6 |  |  | | hsa-miR-3652 | 3.2 | |
| hsa-miR-370-3p | 2.2 | hsa-miR-4688 | 3.8 |  |  | | hsa-miR-370-3p | 3.0 | |
| hsa-miR-377-3p | 2.4 | hsa-miR-4738-3p | 3.7 |  |  | | hsa-miR-377-3p | 3.7 | |
| hsa-miR-379-5p | 2.3 | hsa-miR-4758-5p | 2.6 |  |  | | hsa-miR-379-5p | 4.4 | |
| hsa-miR-381-3p | 2.4 | hsa-miR-493-5p | 2.6 |  |  | | hsa-miR-381-3p | 4.8 | |
| hsa-miR-382-5p | 2.1 | hsa-miR-501-3p | 2.5 |  |  | | hsa-miR-382-5p | 3.5 | |
| hsa-miR-3911 | 2.3 | hsa-miR-501-5p | 2.6 |  |  | | hsa-miR-3911 | 5.1 | |
| hsa-miR-3940-3p | 2.7 | hsa-miR-502-3p | 3.0 |  |  | | hsa-miR-3940-3p | 3.1 | |
| hsa-miR-409-3p | 2.5 | hsa-miR-511-3p | 2.8 |  |  | | hsa-miR-409-3p | 5.5 | |
| hsa-miR-410-3p | 2.2 | hsa-miR-5189-3p | 3.5 |  |  | | hsa-miR-410-3p | 3.7 | |
| hsa-miR-4312 | 2.9 | hsa-miR-518e-5p | 3.2 |  |  | | hsa-miR-4312 | 3.5 | |
| hsa-miR-431-3p | 2.2 | hsa-miR-526b-5p | 3.1 |  |  | | hsa-miR-431-3p | 3.3 | |
| hsa-miR-431-5p | 2.3 | hsa-miR-5571-5p | 2.7 |  |  | | hsa-miR-431-5p | 3.8 | |
| hsa-miR-432-5p | 2.3 | hsa-miR-563 | 2.6 |  |  | | hsa-miR-432-5p | 4.3 | |
| hsa-miR-4430 | 2.3 | hsa-miR-625-5p | 2.5 |  |  | | hsa-miR-4430 | 3.4 | |
| hsa-miR-4462 | 2.2 | hsa-miR-629-3p | 2.3 |  |  | | hsa-miR-4462 | 3.1 | |
| hsa-miR-4651 | 2.5 | hsa-miR-634 | 2.5 |  |  | | hsa-miR-4651 | 3.5 | |
| hsa-miR-4656 | 2.3 | hsa-miR-6511b-5p | 3.5 |  |  | | hsa-miR-4656 | 3.1 | |
| hsa-miR-4666b | 2.8 | hsa-miR-6776-5p | 3.4 |  |  | | hsa-miR-4666b | 3.1 | |
| hsa-miR-4750-3p | 2.7 | hsa-miR-6777-5p | 3.2 |  |  | | hsa-miR-4750-3p | 3.4 | |
| hsa-miR-495-3p | 2.5 | hsa-miR-6778-5p | 3.0 |  |  | | hsa-miR-495-3p | 3.5 | |
| hsa-miR-5196-3p | 2.6 | hsa-miR-6793-5p | 3.4 |  |  | | hsa-miR-5196-3p | 3.2 | |
| hsa-miR-5571-5p | 3.0 | hsa-miR-6824-5p | 3.0 |  |  | | hsa-miR-5571-5p | 3.7 | |
| hsa-miR-563 | 2.6 | hsa-miR-6880-5p | 3.4 |  |  | | hsa-miR-563 | 3.5 | |
| hsa-miR-5699-5p | 2.4 | hsa-miR-760 | 3.5 |  |  | | hsa-miR-5699-5p | 3.3 | |
| hsa-miR-634 | 2.6 | hsa-miR-7845-5p | 3.7 |  |  | | hsa-miR-634 | 3.2 | |
| hsa-miR-6516-3p | 2.2 | hsa-miR-7846-3p | 3.2 |  |  | | hsa-miR-6516-3p | 3.1 | |
| hsa-miR-6732-3p | 2.7 | hsa-miR-8064 | 4.5 |  |  | | hsa-miR-6732-3p | 3.4 | |
| hsa-miR-6756-3p | 2.8 |  |  |  |  | | hsa-miR-6756-3p | 3.2 | |
| hsa-miR-6759-3p | 2.7 |  |  |  |  | | hsa-miR-6759-3p | 3.5 | |
| hsa-miR-6807-5p | 2.5 |  |  |  |  | | hsa-miR-6807-5p | 3.4 | |
| hsa-miR-6834-3p | 2.8 |  |  |  |  | | hsa-miR-6834-3p | 3.3 | |
| hsa-miR-6880-5p | 2.5 |  |  |  |  | | hsa-miR-6880-5p | 3.8 | |
| hsa-miR-6890-3p | 2.7 |  |  |  |  | | hsa-miR-6890-3p | 3.4 | |
| hsa-miR-7108-3p | 2.7 |  |  |  |  | | hsa-miR-7108-3p | 3.2 | |
| hsa-miR-7114-3p | 2.6 |  |  |  |  | | hsa-miR-7114-3p | 3.3 | |
| hsa-miR-7114-5p | 2.5 |  |  |  |  | | hsa-miR-7114-5p | 3.3 | |
| hsa-miR-887-3p | 2.4 |  |  |  |  | | hsa-miR-887-3p | 2.8 | |

Abbreviations: PHH, primary human hepatocyte; KC, Kupffer cell; LSEC, liver sinusoidal endothelial cell; HSC, hepatic stellate cell.
