# Supplementary material for: Human hepatocyte-enriched miRNA-192-3p promotes HBV replication through inhibiting Akt/mTOR signalling by targeting ZNF143 in hepatic cell lines
Source: Emerg Microbes Infect. 2022 Feb 21;11(1):616–28. doi: 10.1080/22221751.2022.2037393 (PMC8865105; doi:10.1080/22221751.2022.2037393)
Supplement: Supplemental Material [file TEMI_A_2037393_SM2028.docx]

**Supplementary** **materials**

**Supplementary methods**

**Cell culture and transfection**

HepG2.2.15 cells harboring integrated dimers of the HBV genome (GenBank accession number U95551) were cultured with 500 μg/mL of G418 (Sigma-Aldrich, Steinheim, Germany). Huh7 cells were grown in Dulbecco’s modified Eagle’s medium as described previously (1). MiRNA mimics, anti-miRNAs or siRNAs were transfected at a concentration of 40nM using Lipofectamine 2000 (Invitrogen, Carlsbad, CA) according to the manufacturer’s instructions. (Qiagen, Hilden, Germany).

**Analysis of HBV replicative capacity *in vitro***

Cell supernatants were collected for HBeAg and HBsAg detection using the Architect System. At 96h post-transfection, cells were harvested and intracellular, encapsulated HBV DNA was extracted and analyzed by Southern blotting analysis as previously described (2).

**Vector construction and luciferase reporter assay**

The regions of HBV core promoter (nt 1648 - 1853), HBV X promoter (nt 1237 - 1375), SP1 promoter (nt 2224 - 2784) and SP2 promoter (nt 2814 - 3123) were amplified from pSM2 plasmid using primers listed in the Supporting Tab. 2. The PCR products were restricted and inserted between MluI and BglII restriction sites into pGL3-basic vector (Promega, Madison, WI), resulting in the luciferase reporter vectors pSP1, pSP2, pCP and pXP, respectively. All clones were subjected to sequencing to verify the correctness of the nucleotide sequences. The primers used for cloning are listed in Supporting table 2 (synthesized by Biomers, Ulm, Germany). For reporter assays, cells were seeded in 24-well plates and cultured for 24 h. 40 nM miRNA was co-transfected with 100 ng of luciferase reporter using Lipofectamine 2000. After 48 h, cells were washed with PBS and resuspended in lysis buffer, followed by detection of luciferase activity in a luminescence reporter gene assay system according to the manufacturer’s instructions (PerkinElmer, Norwalk, CT). All experiments were performed in triplicate, and the results presented are means of three separate experiments.

**Western blot**

72 hours after transfection, cell were harvested for Western blotting analysis. Protein samples were subjected to SDS-PAGE and blotted with primary antibodies against ZNF143, Akt, phosphorylated Akt (p-Akt), mTOR, phosphorylated mTOR (p-mTOR), phosphorylated RB (p-RB, Cell signaling technology, Danvers, MA) and β-actin (Sigma-Aldrich), respectively. Protein bands were visualized using ECL Plus Western blotting detection reagents (Amersham Biosciences, Buckinghamshire, UK), as described previously (2).

**Effect of miR-192-3p on HBV replication in HBV-infected PHHs**

Cryopreserved PHHs purchased from BioreclamationIVT were cultured as previous described (3). In brief, the cells were thawed, washed, and then seeded into collagen I-coated plates. The day after plating, the cells were infected with concentrated HBV particles (collected from HepAD38 supernatant, MOI 200) in Null medium (Williams’ medium E containing B27, Glutamax, Pen Strep, and supplemented with 4% PEG8000). After 20 h, cells were rinsed with PBS and cultured with 5C medium (Null medium supplemented with 5 chemicals: Forskolin, 20 μM; SB431542, 10 μM; IWP2, 0.5 μM; DAPT, 5 μM; and LDN193189, 0.1 μM). Samples were then treated and collected at indicated times as shown in the following flow chart. Briefly, at day 4 post HBV infection, the cells were transfected with 100 nM miR-192-3p mimics or inhibitors with lipo3000. At day 6, 8, 10 post HBV infection, supernatant was collected for HBsAg and HBeAg detection. At day 10 post HBV infection, cells were harvested for intracellular HBV DNA and RNA detection by Realtime qPCR or RT-qPCR.


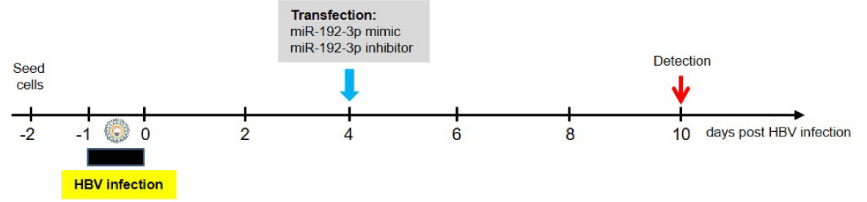


Scheme outlining the HBV infection experiments in PHHs

**References**

1. Li F, Zhou P, Deng W, Wang J, Mao R, Zhang Y, Li J, et al. Serum microRNA-125b correlates with hepatitis B viral replication and liver necroinflammation. Clin Microbiol Infect 2016;22:384 e381-384 e310.

2. Lin Y, Deng W, Pang J, Kemper T, Hu J, Yin J, Zhang J, et al. The microRNA-99 family modulates hepatitis B virus replication by promoting IGF-1R/PI3K/Akt/mTOR/ULK1 signaling-induced autophagy. Cell Microbiol 2017;19.

3. Xiang, C., et al., Long-term functional maintenance of primary human hepatocytes in vitro. Science (New York, N.Y.), 2019. 364(6438): p. 399-402.

**Supplementary figures**


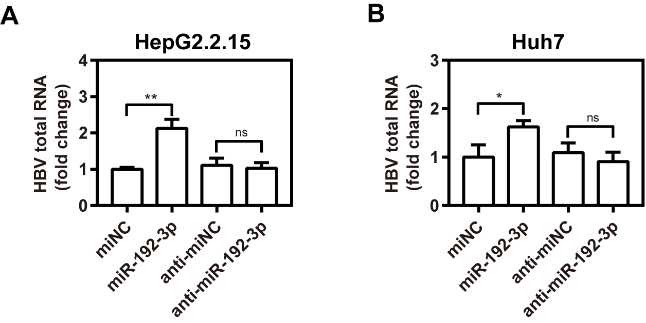


**Figure S1.** **The miR-192-3p increases HBV total RNA level in hepatic cell lines.** HepG2.2.15 cells were transfected with miR-192-3p mimics or inhibitors at 40 nM; Huh7 cells were co-transfected with pSM2 plasmid and miR-192-3p mimics or inhibitors at 40 nM, and harvested. At 72 h post transfection, HBV total RNA level was measured by realtime RT-PCR using specific primers. *, *P* < 0.05; **, *P* < 0.01; ns, no significance.

**
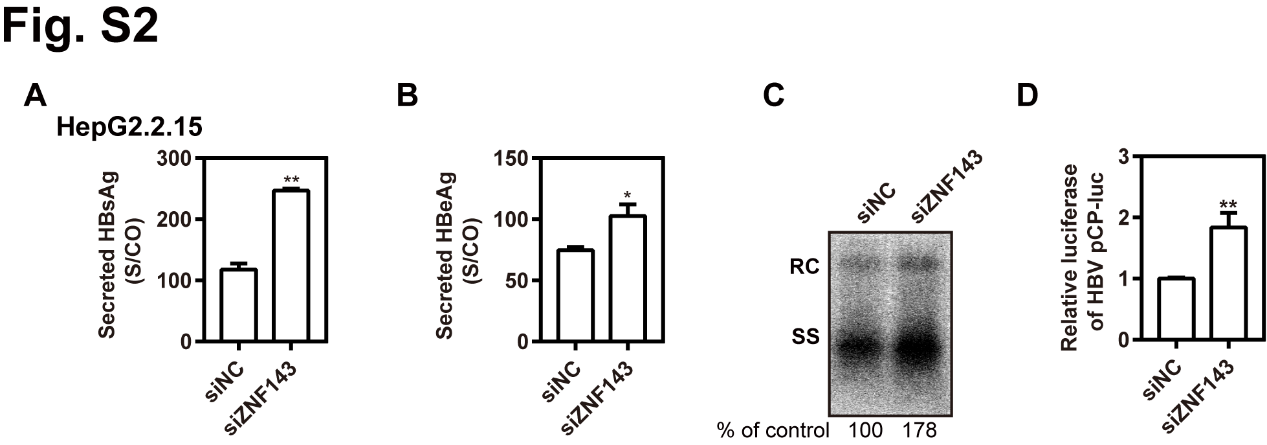
**

**Figure S2. ZNF143 silencing inhibits HBV replication in HepG2.2.15 cells.** HepG2.2.15 cells were transfected with specific siRNAs against ZNF143 (siZNF143) or negative control (siNC) at 40 nM and harvested at 72 h post transfection. Secreted HBsAg (A) and HBeAg (B) levels in culture supernatants were determined by chemiluminescence immunoassay. (C) Encapsidated HBV replicative intermediates were isolated and detected by Southern blotting. (D) HepG2.2.15 cells were co-transfected with siZNF143 and HBV promoter luciferase reporters containing the region of pCP for 48 h with Renilla as an internal control. The data from dual-Glo luciferase report assay were calculated by fold change, and normalized to the siNC samples. *, *P* < 0.05; **, *P* < 0.01.


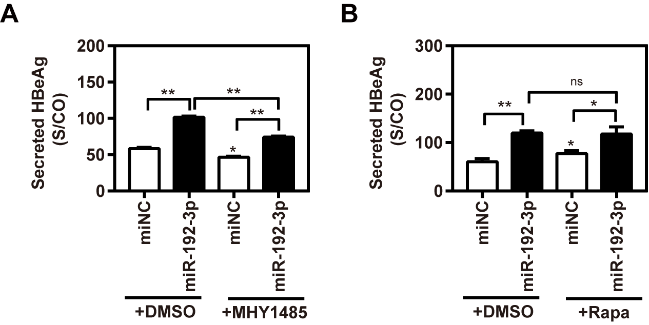


**Figure S3. miR-192 increases HBeAg secretion through inhibiting mTOR signaling in HepG2.2.15 cells.** (A) HepG2.2.15 cells were transfected with specific miR-192-3p mimics or miNC at 40 nM. At 24 h post miRNA transfection, the cells were treated with 2µM MHY 1485 (A) or Rapamycin (B) for 48 h. At 72 h post miRNA transfection. Secreted HBeAg level in culture supernatants was determined by chemiluminescence immunoassay. *, *P* < 0.05; **, *P* < 0.01; ns, no significance.


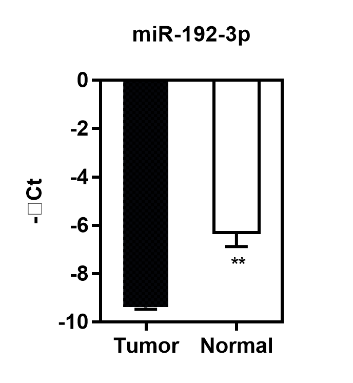


**Figure S4. Hepatic miR-192-3p was significantly higher in normal liver than liver tumor tissue.** **, *P* < 0.01.


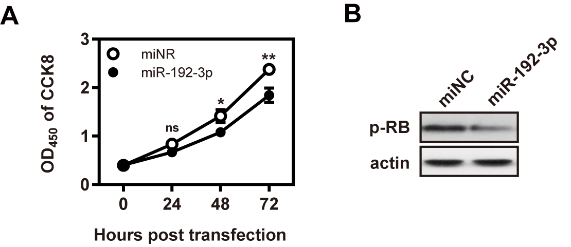


**Figure S5. The miR-192-3p inhibits proliferation of HepG2.2.15.** HepG2.2.15 cells were transfected with miR-192-3p mimics or negative control (miNC). (A) Cell viability was measured by CCK8 assay at 0, 24, 48, or 72 h post transfection. (B) At 72 h post transfection, western blotting analysis was performed to detect the level of phosphorylated RB (p-RB) expression. *, *P* < 0.05; **, *P* < 0.01; ns, no significance.
